# Axial Ligation and Redox Changes at the Cobalt Ion in Cobalamin Bound to Corrinoid Iron-Sulfur Protein (CoFeSP) or in Solution Characterized by XAS and DFT

**DOI:** 10.1371/journal.pone.0158681

**Published:** 2016-07-06

**Authors:** Peer Schrapers, Stefan Mebs, Sebastian Goetzl, Sandra E. Hennig, Holger Dau, Holger Dobbek, Michael Haumann

**Affiliations:** 1 Freie Universität Berlin, Department of Physics, 14195, Berlin, Germany; 2 Humboldt-Universität zu Berlin, Department of Biology, 10115, Berlin, Germany; Argonne National Laboratory, UNITED STATES

## Abstract

A cobalamin (Cbl) cofactor in corrinoid iron-sulfur protein (CoFeSP) is the primary methyl group donor and acceptor in biological carbon oxide conversion along the reductive acetyl-CoA pathway. Changes of the axial coordination of the cobalt ion within the corrin macrocycle upon redox transitions in aqua-, methyl-, and cyano-Cbl bound to CoFeSP or in solution were studied using X-ray absorption spectroscopy (XAS) at the Co K-edge in combination with density functional theory (DFT) calculations, supported by metal content and cobalt redox level quantification with further spectroscopic methods. Calculation of the highly variable pre-edge X-ray absorption features due to core-to-valence (ctv) electronic transitions, XANES shape analysis, and cobalt-ligand bond lengths determination from EXAFS has yielded models for the molecular and electronic structures of the cobalt sites. This suggested the absence of a ligand at cobalt in CoFeSP in α-position where the dimethylbenzimidazole (dmb) base of the cofactor is bound in Cbl in solution. As main species, (dmb)Co^III^(OH_2_), (dmb)Co^II^(OH_2_), and (dmb)Co^III^(CH_3_) sites for solution Cbl and Co^III^(OH_2_), Co^II^(OH_2_), and Co^III^(CH_3_) sites in CoFeSP-Cbl were identified. Our data support binding of a serine residue from the reductive-activator protein (RACo) of CoFeSP to the cobalt ion in the CoFeSP-RACo protein complex that stabilizes Co(II). The absence of an α-ligand at cobalt not only tunes the redox potential of the cobalamin cofactor into the physiological range, but is also important for CoFeSP reactivation.

## Introduction

The cobalamin cofactor (Cbl, also denoted vitamin B_12_) since its discovery in 1925 has attracted much research interest [1–4]. Cbl is essential for all mammals [5] and in bacteria it is involved in carbon oxide (CO_x_) conversion pathways related to potential renewable energy applications [6, 7]. Anaerobic CO_2_ reduction along the bacterial Wood-Ljungdahl pathway includes several unique enzymes [8, 9]. The corrinoid iron-sulfur protein (CoFeSP) carries a Cbl cofactor [10, 11] and shuttles a methyl group from methyl-transferase bound methyl-tetrahydrofolate to acetyl-CoA synthase. The latter enzyme, after receiving a CO group derived from CO_2_ reduction by carbon monoxide dehydrogenase, synthesizes acetyl-CoA for many metabolic reactions [12]. CoFeSP alternates in the methyl transfer cycle between Co(III)-CH_3_ and Co(I) states [13]. The Co(I) state is prone to oxidative inactivation generating Co(II), which can be reductively reactivated in an ATP-dependent reaction catalyzed by the reductive-activator protein (RACo) [14–16]. Redox and ligation changes at cobalt in Cbl in the CoFeSP-RACo system thus are essential in the CO_x_ conversion pathway.

**Fig 1:**
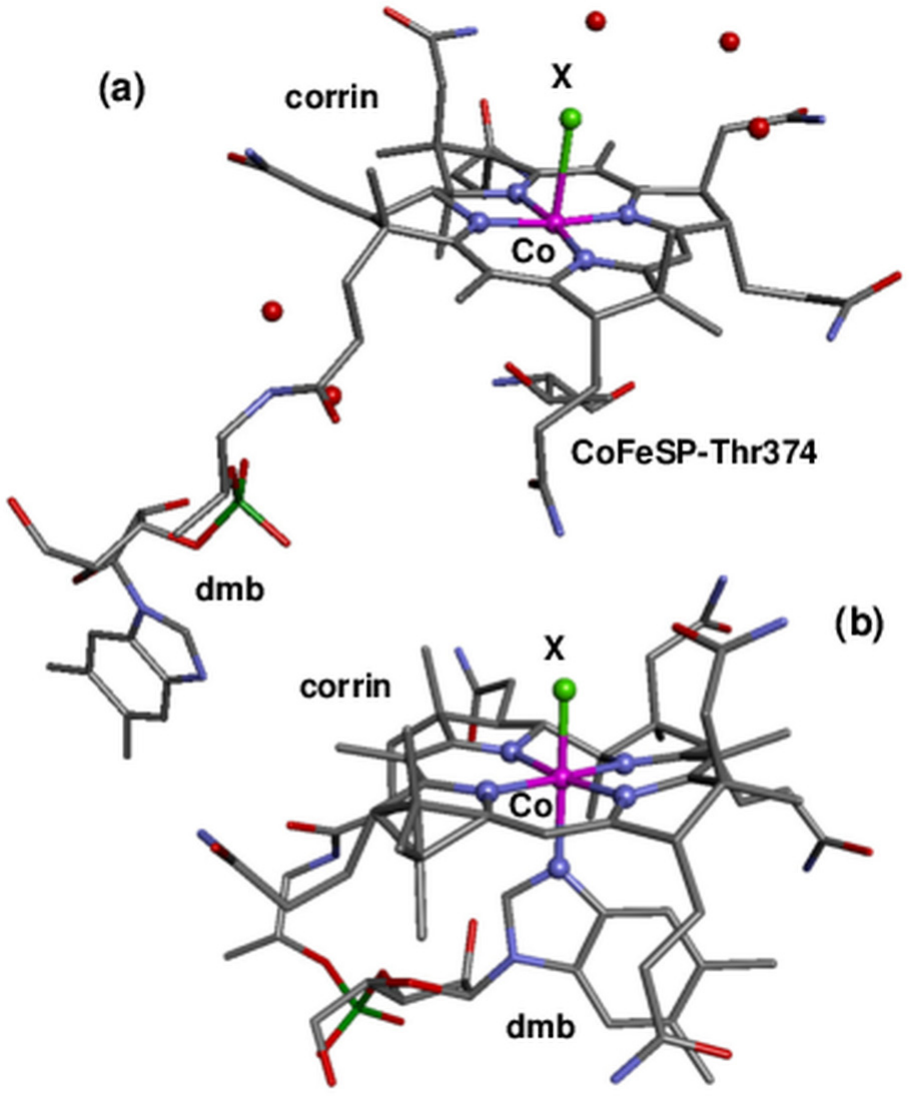
Cobalamin crystal structures. (a) Structure of the Cbl cofactor in CoFeSP enzyme (PDB entry 2H9A, 1.9 Å resolution [12]) showing a base-off configuration (dmb ligand not bound to cobalt in α-position). Ligand X at the ß-position (light green) at cobalt can be absent or can be a water species, a methyl group, or an oxygen from the side chain of RACo-Ser398 in the CoFeSP-RACo complex [14]; red balls show resolved water molecules. (b) Structure of Cbl in base-on configuration [21]; X can be a water, cyanide, or methyl species. Color code: magenta, Co; blue, N; red, O; grey, C; dark green, P; protons were omitted for clarity.

Cobalamin is among the most complex non-polymeric compounds in nature and consists of a unique corrin hetero-macrocycle binding a central cobalt ion by four equatorial nitrogen ligands [17]. Two axial cobalt ligands (α and ß) may be bound in addition. The α-ligand in Cbl in solution or in prototypic Cbl-proteins in the so-called base-on configuration is the nitrogen atom of a dimethylbenzimidazole (dmb) group connected to the corrin ring (Fig 1). Replacement of the dmb ligand (base-off) by a water species or by other amino acids occurs in many proteins [1–3]. Crystal structures of isolated CoFeSP and of the protein in complex with RACo or methyl transferase have been reported [12, 14, 16, 18, 19]. In all CoFeSP structures, the dmb group is folded away from the corrin so that the α-site apparently is vacant (Fig 1). However, it could also be occupied by a crystallographically less visible (disordered) water species or even by the hydroxyl group of a nearby threonine residue modeled at about 3.5 Å to cobalt in the structures. The ß-site in CoFeSP-Cbl can be occupied by a water species (AqCbl), a methyl group (MeCbl) [20], or may be vacant (Fig 1). In the CoFeSP-RACo protein complex, binding of the hydroxyl group of a serine (Ser 398) of RACo to cobalt at the ß-position has been shown [14–16].

Binding of the axial ligands is closely related to the cobalt oxidation state [4]. In Cbl, both in solution and bound to proteins, the formal Co(I), Co(II), and Co(III) states are associated with low-spin (3*d*^8^, 3*d*^7^, 3*d*^6^) valence electron configurations [22–24]. Only Co(II) thus is EPR active. A decrease of the oxidation state may be accompanied by a decreasing number of axial ligands, meaning that (L = ligand) (L α)Co^III^(Lß), Co^II^(Lß) or (L α)Co^II^, and Co^I^ species may prevail [24, 25], but in protein environments deviations from such configurations may occur. Control of the axial cobalt ligation in group-transferring Cbl-enzymes such as CoFeSP is important in the reactions. However, relating the redox state to the axial ligation of cobalt can be difficult both by crystallography and spectroscopy. For example, in solution Cbl mixtures of base-on/off states may occur, in crystal structures of Cbl-proteins axial ligands may be unresolved, or certain spectroscopic methods do not provide structural and electronic parameters or only for selected cobalt redox states. Further insight in the cobalt site structures in the CoFeSP-Cbl-RACo system is required to understand the interplay of protein-protein interactions, redox transitions, axial ligand exchange, and methyl group transfer.

Here, we employed X-ray absorption spectroscopy (XAS) at the Co K-edge in combination with density functional theory (DFT) to study redox and coordination changes at cobalt in CoFeSP-Cbl in comparison to Cbl in solution. XAS in principle facilitates oxidation state, metal-ligand bond lengths, and site symmetry determination for solution and protein systems and can be applied to all spin and oxidation states of metal sites [26–29]. In particular the XAS features due to resonant 1*s* electron excitation into unoccupied valence levels (for example with Co(3*d*) character) in the so-called pre-edge absorption spectral region (core-to-valence transitions, ctv), which can be calculated by DFT [30–33], are sensitive to the molecular and electronic structure of the Cbl cofactor [34–39]. Pronounced alterations of the ctv spectra upon changes at cobalt were observed, which were reproduced by the computational approach. Combination of experimental and theoretical analyses has established relations between the X-ray spectroscopic features and the redox state and axial ligation at the cobalt centers, thereby providing structural models for the AqCbl and MeCbl cofactors in CoFeSP and in the CoFeSP-RACo protein complex.

## Materials and Methods

### Sample preparation

CoFeSP and RACo proteins from *Carboxydothermus hydrogenoformans* were heterologously overexpressed in *Escherichia coli* following previously established protocols [15, 16, 19] and protein purification and biochemical treatments were performed under anoxic conditions in 95% N_2_ and 5% H_2_ atmosphere at room temperature in a glove box. Synthetic cobalamin (denoted AqCbl^ox^, CNCbl^ox^, and MeCbl^ox^) containing Co(III) was purchased from Sigma-Aldrich, all chemicals were at least analysis grade. Purified CoFeSP (25 μM) was reconstituted in 20 mM TRIS-HCl buffer (pH 8.0) with synthetic AqCbl^ox^ or MeCbl^ox^ (40 μM) by overnight incubation at 25°C, subsequently unbound cofactor was removed and the protein concentrated using Vivaspin 500 concentrators (10 kDa cut-off). CoFeSP-AqCbl^ox^ and CoFeSP-MeCbl^ox^ samples for XAS contained 1.0±0.1 mM protein as determined by the Bradford method [40]. A glass-forming agent was not present in the XAS samples. Titanium(III)-citrate (2 mM) was added to CoFeSP-Cbl^ox^ samples for cofactor reduction (red). The CoFeSP-AqCbl-RACo protein complex was prepared according to the previously reported protocol [14–16]. Solution samples of synthetic cobalamins (7 mM) were prepared by solvation of AqCbl^ox^, CNCbl^ox^, and MeCbl^ox^ powders in 20 mM TRIS-HCl buffer (pH 8.0) and chemical reduction was achieved by addition of sodium dithionite (100 mM) to obtain CNCbl^red^ or titanium-citrate (40 mM) to obtain AqCbl^red^ samples. Aliquots of samples (50 μL) were loaded into Kapton-covered acrylic glass holders for XAS and frozen in liquid nitrogen. Optical absorption spectra of samples using 2 μl aliquots were recorded on a Specord 50 Plus instrument (Analytik Jena, Germany).

### Total reflection X-ray fluorescence analysis

TXRF [41] was performed for metal content determination in protein samples using a PicoFox instrument (Bruker, Berlin, Germany). Protein samples were mixed (v/v 2:1) with a gallium concentration standard (Sigma, 50 mg/L) and three measurements were carried out per sample using 5 μl aliquots.

### X-ray absorption spectroscopy

XAS at the Co K-edge was performed at the bending-magnet beamline KMC-1 at BESSY (Helmholtz-Center for Materials and Energy Berlin) with the storage ring operated in top-up mode (250 mA). The excitation energy was tuned by a Si[111] double-crystal monochromator. Kα-fluorescence-detected XAS spectra were collected using an energy-resolving 13-element germanium detector (Canberra) on samples held in a liquid-helium cryostat (Oxford) at 20 K. The detector was shielded against scattered X-rays by a 10 μm iron foil. The K-edge inflection point at 7709 eV of a simultaneously measured cobalt metal foil was used for calibration of the energy axis. Detector deadtime corrected XAS spectra (scan duration ~30 min) were averaged (up to 9 scans, 2 scans per sample spot) for signal-to-noise ratio improvement. No radiation induced spectral changes (i.e. in the XANES) were observed for increasing XAS scan numbers on single sample spots. XAS data processing was carried out as previously described [27] to yield normalized XANES and EXAFS spectra. Simulation of *k*^3^-weighted EXAFS spectra in k-space was carried out using the in-house software SimX and phase functions calculated with FEFF7.0 (S_0_^2^ = 0.85) [42, 43]. In the fits the number of C-atoms was set to the values corresponding to the corrin ring. Fourier-transforms of EXAFS spectra were calculated for *k* = 1.8–12.2 Å^-1^ using cosine windows extending over 10% of both *k*-range ends. The XANES pre-edge features were extracted by polynomial spline subtraction with the program XANDA [44]. Multiple-scattering theory simulations of K-edge spectra were performed with the FEFF9.0 code [45] using model structures based on the cobalamin crystal structure in CoFeSP (PDB entry 2H9A, 1.9 Å resolution [12]); for details see the Supporting Information (Fig C in S1 File).

### Density functional theory calculations

Starting geometries for DFT were derived from cobalamin crystal structures, in which the axial cobalt ligands were modified and structures were truncated to minimize calculation times (Fig 1; the nucleotide loop and amide side chains of Cbl were removed, in base-off models the dmb was removed, in base-on models a benzimidazole group mimicked the dmb ligand, as further ligands, OH^-^, H_2_O, CH_3_^-^, or CN^-^ groups were added). The total charge and spin multiplicity of the models was set to the desired low-spin cobalt oxidation state [21, 46]. The model structures were geometry-optimized using the Gaussian09 package [47], the B3LYP functional [48], and a triple-zeta-valence-plus-polarization basis set (TZVP) [49] on the Soroban computer cluster of the Freie Universität Berlin. The theoretical approach was selected because it provided spectra which near-quantitatively agree both in absolute and relative shapes with the experimental data, besides of showing good agreement between experimental and calculated site geometries. Natural population analysis (NPA) charges [50] were calculated with the NBO-5 program [51]. The pre-edge features (ctv) in the XANES were calculated by DFT using the ORCA program [52, 53] on the basis of the geometry-optimized model structures as previously described [30–33]. The calculated ten ctv transitions (sticks) at lowest energies were broadened by Gaussian functions (FWHM 2.5 eV), 158.7 eV shifted on the energy axis, and their amplitudes were scaled (x900) for comparison with experimental ctv spectra.

## Results

### Cofactor content and oxidation state

Cobalamin (Cbl) species were investigated when bound to the CoFeSP enzyme, in the CoFeSP-RACo protein complex, and in solution samples serving as reference materials. The expected axial cobalt ligations in the samples included dimethylbenzimidazole (dmb) or water (Aq) species at the α position (occupied by the dmb ligand in crystalline Cbl) or Aq, cyanide (CN), or methyl (Me) group species at the ß position (opposite to the dmb ligand). CoFeSP-Cbl and solution Cbl samples in the oxidized state (ox) and after chemical reduction (red) were compared. We denote the various redox and axial ligation species of cobalt as (α-ligand)Co^x^(ß-ligand) (x = valence state) in the following.

The cobalt concentrations in the Cbl-reconstituted protein samples were determined by TXRF, which on average yielded 0.8±0.1 Co ions per CoFeSP containing AqCbl or MeCbl (Table 1). This suggested close to stoichiometric reconstitution of CoFeSP with the cofactors. The mean amount of 3.5±0.5 Fe ions per CoFeSP protein was in reasonable agreement with the near-quantitative presence of the [4Fe4S] cluster in CoFeSP. The mean Fe to Co ratio was 4.6±0.2, which for 4 Fe ions in the [4Fe4S] cluster per protein, suggested ~0.85 Co ions per CoFeSP, in agreement with the protein to cobalt ratios. The increased Fe to Co ratio of 6.5 in the CoFeSP-AqCbl-RACo sample was in good agreement with two additional Fe ions in the sample compared to CoFeSP-AqCbl, due to the presence of close to one RACo protein containing a [2Fe2S] cluster per CoFeSP.

**Table 1:** Metal content and cobalt oxidation state in the CoFeSP samples^a^.

sample	Fe [mM]	Co [mM]	Fe/Co	Co(I) [%]	Co(II)^b^ [%]	Co(III)^d^ [%]
CoFeSP-AqCbl^ox^	3.7	0.8	4.6	0	30	70
CoFeSP-AqCbl^red^	2.3	0.5	4.6	30^c^	70	0
CoFeSP-AqCbl-RACo	3.9	0.6	6.5	0	85	15
CoFeSP-MeCbl^ox^	3.3	0.7	4.7	0	0	100

^a^Protein concentrations for CoFeSP-AqCbl^ox^ and -MeCbl^ox^ were 1.0±0.1 mM. Metal concentrations were determined by TXRF (error ±0.1 mM)

^b^Co(II) contents were determined by EPR (Fig B in S1 File, error ±10%)

^c^Co(I) contents were estimated from optical absorption spectra (Fig A in S1 File)

^d^Co(III) contents agree with Co(II)/Co(I) contents and data in Figs A and B in S1 File.

Optical absorption spectra (Fig A in S1 File) of the solution Cbl samples confirmed the expected quantitative presence of Co(III) in AqCbl^ox^, CNCbl^ox^, and MeCbl^ox^, and showed mostly Co(II) in AqCbl^red^ and a Co(II) species in CNCbl^red^. For the protein samples, the absorption spectra (Fig A in S1 File) indicated the expected Co(III) in the cofactor in CoFeSP-MeCbl^ox^, suggested dominance of Co(III) in oxidized CoFeSP-AqCbl and of Co(II) in CoFeSP-AqCbl-RACo, and showed preferentially Co(II) in CoFeSP-AqCbl^red^ with minor (~30%) Co(I) amounts only in this sample. Electron paramagnetic resonance spectroscopy (EPR) detecting only the Co(II)-containing cofactor was used to quantify the relative Co(II) contents in the protein samples (Fig B in S1 File). This showed that CoFeSP-AqCbl^ox^ contained ~30% Co(II) and, considering also the optical spectra, ~70% Co(III), CoFeSP-AqCbl^red^ contained ~70% Co(II), and CoFeSP-AqCbl-RACo near-quantitative amounts of Co(II) (~85%) (Table 1). The altered EPR signal shape of CoFeSP-AqCbl-RACo (Fig B in S1 File) also suggested near-stoichiometric RACo binding to CoFeSP [15, 19], in agreement with the TXRF data.

### EXAFS on the Cbl systems

**Fig 2:**
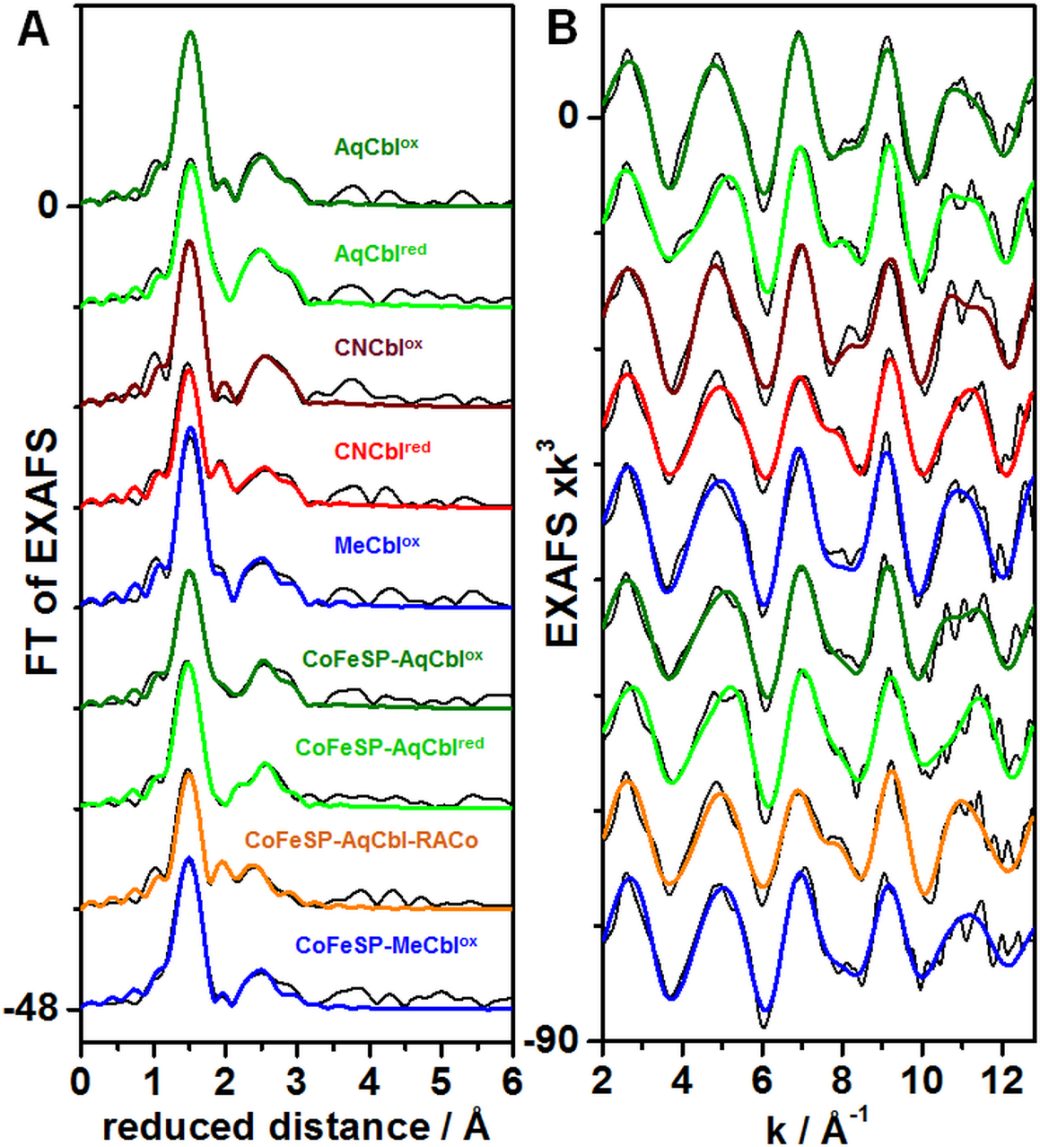
EXAFS spectra of cobalamin systems. Panel (A) shows Fourier-transforms (FTs) of the EXAFS oscillations in panel (B) for indicated solution Cbl or CoFeSP-Cbl samples. Black lines, experimental data; coloured lines, simulations with parameters in Table 2 (fits 2, 5, 7, 10, 12, 14, 16, 19, 21); spectra in (A) and (B) were vertically shifted for comparison.

Simulation of EXAFS spectra facilitates determination of interatomic distances such as the cobalt-ligand bond lengths with ~0.02 Å precision in favorable cases. Visual inspection of the EXAFS spectra of the Cbl and CoFeSP-Cbl samples revealed a dominant Fourier-transform (FT) peak due to Co-C/N/O bonds from the corrin ring and the axial ligands and smaller features at larger distances mostly due to second-sphere Co-C_corrin_ interactions (Fig 2). The fit analysis (Table 2) revealed typical bond lengths (~1.87 Å) of the equatorial Co-N_corrin_ ligands in the AqCbl^ox^, CNCbl^ox^, and MeCbl^ox^ solution samples, which were only ~0.02 Å elongated in AqCbl^red^ and CNCbl^red^. The second-sphere EXAFS features were well described by a mean Co-C_corrin_ distance of ~2.9 Å and a multiple-scattering contribution with an apparent N-C distance in the corrin ring of ~1.4 Å. These Co-N/C_corrin_ distances are in agreement with Cbl crystal structures [54–57] and earlier XAS data [34, 36]. Axial cobalt ligands also were discernable in the EXAFS. For AqCbl^ox^, the dmb (α) and water (ß) ligands showed relatively similar (1.96±0.08 Å) bond lengths at cobalt, attributed to a slightly longer Co-N and shorter Co-O bond [56]. Both bonds were ~0.3 Å elongated in AqCbl^red^ (Table 2). For CNCbl^ox^, the Co-C bond was ~0.04 Å shorter than the Co-N_corrin_ bonds as in crystalline CNCbl^ox^ [55] and the Co-N_dmb_ bond was similar to AqCbl^ox^. Lower coordination numbers and elongated axial bonds (~2.13 Å) in CNCbl^red^ suggested that one ligand possibly was detached. For MeCbl^ox^, the longer and shorter axial bonds likely were attributed to the dmb (~2.20 Å) and CH_3_ (~1.95 Å) ligands [54]. Overall, the fit results showed that axial cobalt ligation changes dominated the EXAFS spectral variations.

**Table 2:** EXAFS simulation parameters^a^.

sample	fit	Co-N	Co-C/N/O	Co-C	R_F_
		N / R / 2σ^2^	N / R / 2σ^2^	N / R / 2σ^2^	[%]
AqCbl^ox^	1	4* / 1.87 / 3*	1.8 / 1.96 / 3*	11* / 2.88 (1.44) / 11^#^	8.1
	2	4* / 1.88 / 3*	0.9 / 1.92 / 3* 0.8 / 1.97 / 3*	11* / 2.89 (1.42) / 10^#^	8.0
AqCbl^red^	3	4* / 1.89 / 3*		11* / 2.90 (1.34) / 8^#^	17.1
	4	4* / 1.89 / 3*	0.9 / 2.29 / 3*	11* / 2.91 (1.37) / 8^#^	13.6
	5	4* / 1.89 / 3*	1.1 / 2.30 / 3* 1.1 / 2.47 / 3*	11* / 2.91 (1.41) / 8^#^	8.4
CNCbl^ox^	6	4* / 1.87 / 3*	1.5 / 1.97 / 3*	11* / 2.87 (1.38) / 10^#^	16.5
	7	4* / 1.89 / 3*	0.8 / 1.85 / 3* 1.2 / 2.05 / 3*	11* / 2.88 (1.39) / 9^#^	12.1
CNCbl^red^	8	4* / 1.87 / 3*		11* / 2.90 (1.40) / 12^#^	19.6
	9	4* / 1.88 / 3*	0.9 / 2.14 / 3*	11* / 2.91 (1.39) / 12^#^	12.3
	10	4* / 1.88 / 3*	1.0 / 2.14 / 3* 0.5 / 2.54 / 3*	11* / 2.91 (1.41) / 13^#^	11.6
MeCbl^ox^	11	4* / 1.89 / 3*	1.3 / 1.93 / 3*	11* / 2.89 (1.43) / 11^#^	10.5
	12	4* / 1.89 / 3*	1.2 / 1.95 / 3* 0.7 / 2.21 / 3*	11* / 2.90 (1.42) / 10^#^	7.3
CoFeSP-AqCbl^ox^	13	4* / 1.88 / 3*	0.7 / 2.01 / 3*	11* / 2.91 (1.41) / 12^#^	10.4
	14	4* / 1.88 / 3*	0.6 / 2.02 / 3* 0.4 / 2.29 / 3*	11* / 2.91 (1.41) / 11^#^	8.5
CoFeSP-AqCbl^red^	15	4* / 1.85 / 3*		11* / 2.88 (1.44) / 16^#^	19.0
	16	4* / 1.86 / 3*	0.6 / 2.31 / 3*	11* / 2.88 (1.44) / 15^#^	14.4
	17	4* / 1.86 / 3*	0.3 / 1.96 / 3* 0.8 / 2.33 / 3*	11* / 2.89 (1.45) / 14^#^	12.8
CoFeSP-AqCbl-RACo	18	4* / 1.88 / 3*	0.8 / 2.12 / 3*	11* / 2.89 (1.34) / 13^#^	19.2
	19	4* / 1.88 / 3*	0.8 / 2.10 / 3* 0.4 / 2.53 / 3*	11* / 2.87 (1.41) / 19^#^	8.4
CoFeSP-MeCbl^ox^	20	4* / 1.87 / 3*	1.2 / 2.00 / 3*	11* / 2.90 (1.42) / 15^#^	14.5
	21	4* / 1.87 / 3*	1.2 / 2.01 / 3* 0.2 / 2.50 / 3*	11* / 2.89 (1.44) / 16^#^	12.5

^a^Data refer to EXAFS spectra in Fig 2. *N*, coordination number per Co ion; *R*, interatomic distance in Å (i.e. cobalt-ligand bond length); 2σ^2^, Debye-Waller parameter in x10^-3^ Å^2^; *R*_F_, fit error sum (calculated for reduced distances of 1–3 Å [27], R_F_ represents the mean root square deviation in % between the experimental Fourier-isolated k-space EXAFS spectrum in the given reduced-distance range of the fit and the fit curve)

*parameters that were fixed at given physically reasonable values in the fits

^#^2σ^2^ was coupled to yield the same values for the ~2.9 Å Co-C shell (N_Co-C_ was set to the crystallographic distances in the ~2.9–3.3 Å range, the Debye-Waller factor reflects this distance distribution with more emphasis on the 8 shorter Co-C distances).

A further Co-N-C multiple-scattering shell with the same *N* and 2σ^2^ values as for the Co-C shell was included in the fits (apparent N-C distances given in parenthesis). The 2σ^2^ values for the Co-N and Co-C/N/O shells were chosen to provide best fit results. Two lines for a given coordination shell mean that both distances were included in the respective fit. We note that splitting of the axial ligation shells in the fit procedure is tentative due to the ~0.1 Å distance discrimination limit of our *k* = 13 Å^-1^ EXAFS data [58]. We note that the small *N*-values of the second Co-C/N/O shell with relatively long distances for CoFeSP-MeCbl (fit 19) and CoFeSP-RACo (fit 21) may not be significant and suggest dominance of 5-coordinated cobalt sites (see Fig F in S1 File).

The CoFeSP-Cbl samples showed similar Co-N/C_corrin_ distances as found for solution Cbl in the EXAFS fits (Table 2), revealing the integrity of the base-off cofactor in the reconstituted protein [18, 19]. For CoFeSP-AqCbl^ox^, lower coordination numbers of the axial ligands compared to solution AqCbl^ox^ suggested only one axial ligand. Two detectable Co-O bond lengths were attributed to a larger contribution (~2.0 Å) from 5-coordinated Co(III) and a smaller contribution (~2.3 Å) from 5-coordinated Co(II). CoFeSP-AqCbl^red^ showed significantly (~0.02 Å) shorter Co-N_corrin_ bonds compared to CoFeSP-AqCbl^ox^, presumably due to the minor Co(I) contribution, and predominance of one axial ~2.3 Å bond, attributed to a water species at Co(II). CoFeSP-AqCbl-RACo revealed only one significant short axial ligand bond (~2.1 Å); a longer interaction (~2.5 Å) showed a small and possibly insignificant coordination number (Fig F in S1 File). The short bond may reflect the Co-O_Ser_ interaction in the CoFeSP-RACo complex. CoFeSP-MeCbl^ox^ revealed only one axial ligand (~2.0 Å) due to the Co(III)-CH_3_ interaction, which was slightly longer than in solution MeCbl^ox^ (Table 2).

### XANES spectral analysis

**Fig 3:**
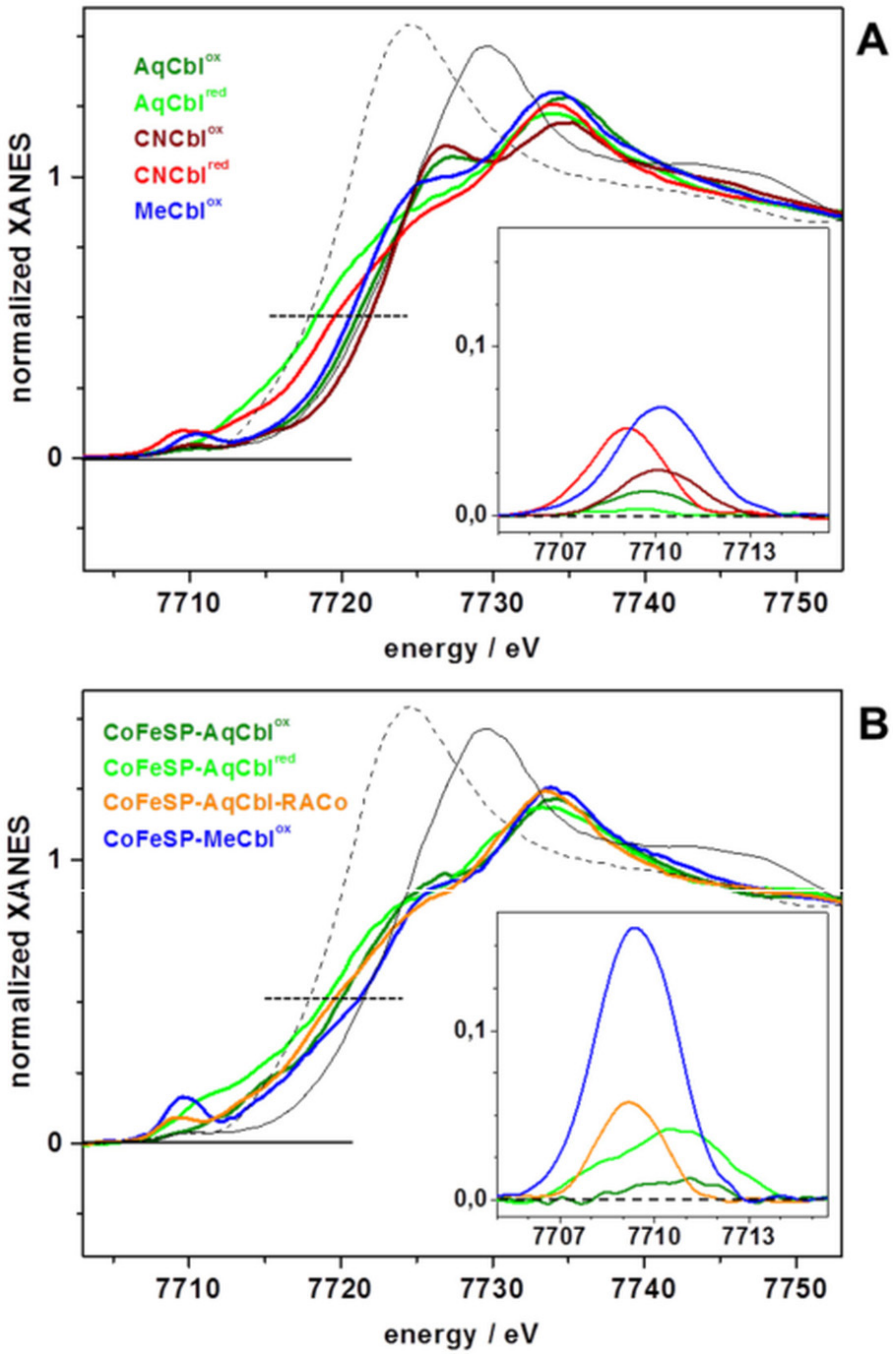
Cobalt XANES spectra. (A) Indicated Cbl solution samples, (B) CoFeSP-Cbl samples. Dotted lines mark edge half-height. Spectra of Co^III^_2_O_3_ (solid black line) and Co^II^O (dashed black line) are shown for comparison in (A) and (B). Inset: Isolated pre-edge (core-to-valence, ctv) absorption features after subtraction of a smooth edge rise background (not shown) from the XANES spectra. For XANES spectra of further cobalt reference compounds see Fig C in S1 File.

**Fig 4:**
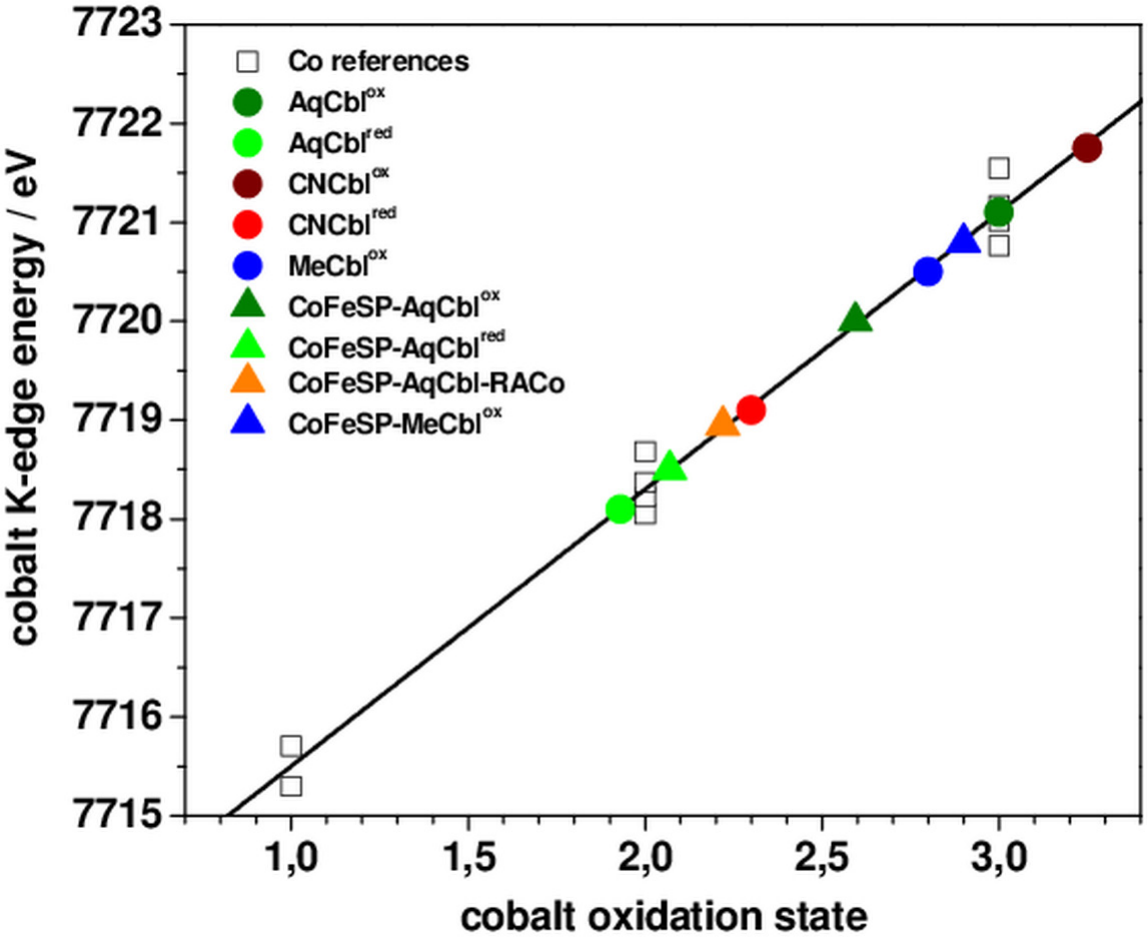
Cobalt K-edge energies. Shown are K-edge energies (at 50% level) of XANES spectra in Fig 3 of Cbl and CoFeSP-Cbl samples (colored symbols) and of cobalt reference compounds (Fig C in S1 File) containing Co(I), Co(II), or Co(III) (open squares). Black line, linear regression to the reference data (E_K-edge_ = 7712.77 eV + 2.76 eV * x, x = cobalt oxidation state); data points for solution Cbl and CoFeSP-Cbl were placed on the fit curve according to their K-edge energies. (For K-edge energies from XANES simulations see Figs D and E in S1 File.)

The XANES spectrum is sensitive to the spin and oxidation state of the metal, as well as to the chemical nature and symmetry of its ligands. The XANES of the solution Cbl and CoFeSP-Cbl samples revealed overall similar shapes (Fig 3), as explained by the spectral dominance of the equatorial N_corrin_ ligands at cobalt. The significantly different K-edge energies (Fig 4) thus likely were related to the cobalt redox and axial ligation changes. Reference K-edge energies for the cobalt redox states species were derived from synthetic complexes (Fig C in S1 File) and were determined as ~7715.5 eV for Co(I), ~7718.3 eV for Co(II), and ~7721.1 eV for Co(III) species, revealing a ~2.8 eV edge energy increase per single-electron cobalt oxidation (Fig 4). All Cbl and CoFeSP-Cbl samples showed K-edge energies in the Co(II) to Co(III) region, well above the Co(I) level (Fig 4). The K-edge energies for MeCbl^ox^, AqCbl^ox^, and CNCbl^ox^ were centered around Co(III), with AqCbl^ox^ located at the mean Co(III) energy and a ~1 eV difference between CNCbl^ox^ and MeCbl^ox^. The ~2.8 eV lower K-edge energies for AqCbl^red^ and CNCbl^red^ sugegsted near-quantitative Co(II) contents. For the protein samples, the K-edge energy of CoFeSP-MeCbl^ox^ was closest to the Co(III) level, but the edge shape differed strongly from solution MeCbl^ox^ (Figs 3 and 4). The edge energy for CoFeSP-AqCbl^ox^ was lower than the mean Co(III) level due to Co(II) admixture and a ~1.5 eV lower edge energy for CoFeSP-AqCbl^red^ reflected the increased Co(II) content. The K-edge energy for CoFeSP-AqCbl-RACo was close to the Co(II) level, but the different edge shape compared to CoFeSP-AqCbl^red^ suggested a coordination change at cobalt.

Qualitative multiple-scattering K-edge simulations on structural models for the cobalt sites (Fig D in S1 File) fairly reproduced the experimental K-edge shape and energy differences between AqCbl^ox^, CNCbl^ox^, and MeCbl^ox^ (base-on octahedral cobalt sites) (Fig E in S1 File). Simulations for the base-off sites in CoFeSP-Cbl showed that replacement of the dmb by a water ligand in 6-coordinated sites results in lower edge energies compared to the base-on structures for AqCbl^ox^ and MeCbl^ox^ similar to the experimental data, α-ligand removal decreased the edge energy compared to the 6-coordinated sites by ~1 eV, elongation of the axial bond as in CoFeSP-AqCbl^red^ resulted in a small (~0.5 eV) edge energy decrease, and the edge energy of a square-planar cobalt site was close to the Co(II) level. These results suggested that the lowered edge energies in the CoFsSP-Cbl^ox^ compared to the solution Cbl^ox^ samples at least in part were explained by loss of one axial ligand and the K-edges of CoFeSP-AqCbl^red^ and the CoFeSP-Cbl-RACo complex reflected a different axial ligand.

### The XANES pre-edge feature

**Fig 5:**
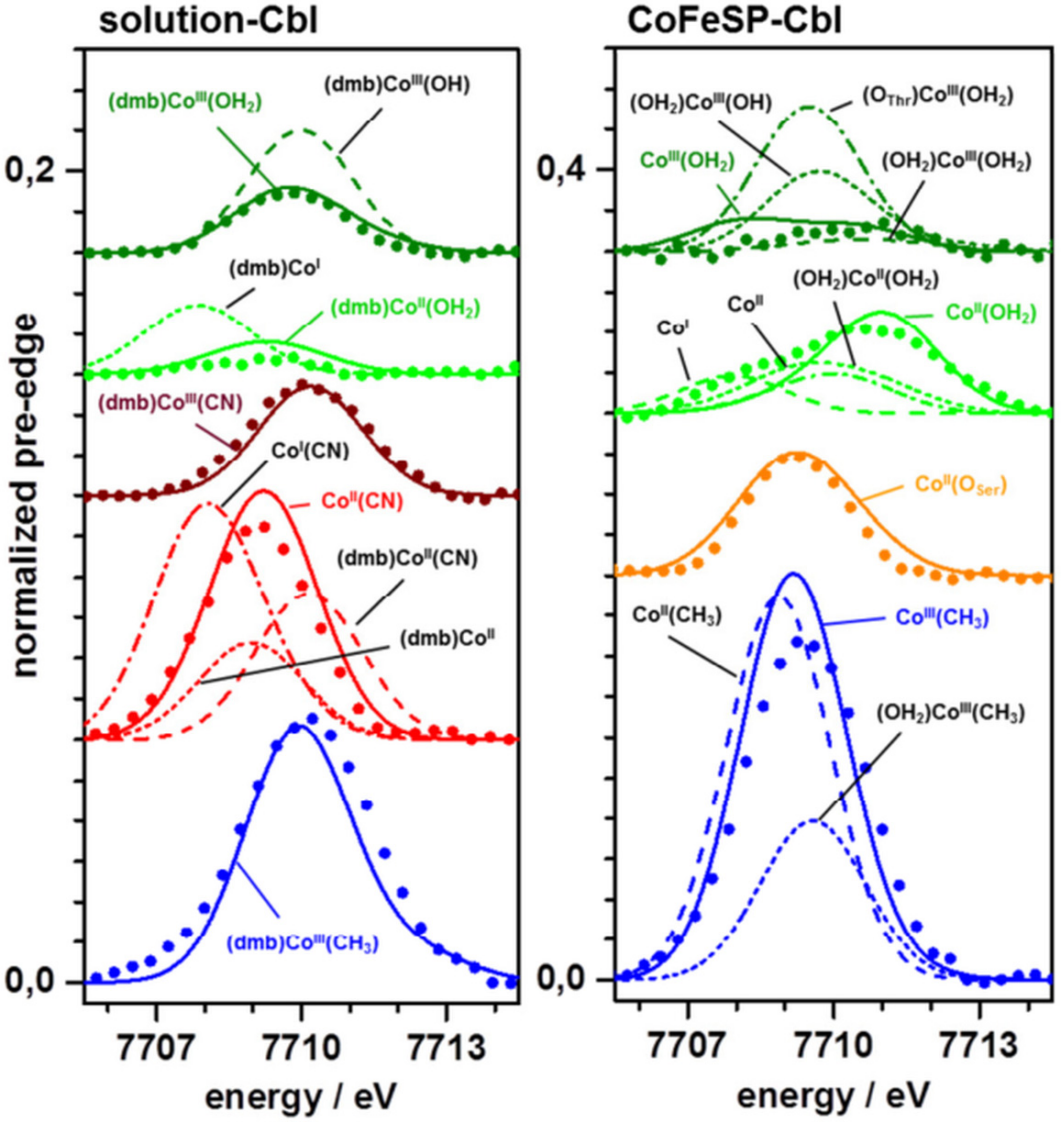
Comparison of DFT calculated and experimental ctv features. Lines, spectra from DFT; dots, experimental data (Fig 3); spectra were vertically shifted for comparison; note the doubled y-scale in (B). Calculated spectra represent the indicated model structures; solid lines and coloured annotations denote calculated spectra for the indicated structures, which show superior agreement with the experimental data (broken lines show calculation results less in agreement with the experimental data).

The pre-edge absorption in the K-edge reflects resonant 1*s* electron excitation into unoccupied valence levels with (partial) Co(3*d*) character (core-to-valence transitions, ctv). Pronounced differences in the ctv spectra were observed between the Cbl systems (Fig 3, insets). The small ctv feature in AqCbl^ox^ was further decreased in AqCbl^red^. A larger ctv amplitude for CNCbl^ox^ compared to AqCbl^ox^ was further increased in CNCbl^red^. MeCbl^ox^ showed the largest ctv feature among the solution samples. An almost negligible ctv feature was observed for CoFeSP-AqCbl^ox^. CoFeSP-AqCbl^red^ showed a much larger amplitude at higher energies and broader envelope of the ctv feature. Also CoFeSP-AqCbl-RACo showed a ctv amplitude increase, but a shift to lower energies. CoFeSP-MeCbl^ox^ exhibited by far the largest ctv feature, exceeding that of solution MeCbl^ox^.Density functional theory (DFT) was employed to generate geometry-optimized model structures of the cobalt sites and to calculate ctv features on their basis (Fig 5). The (dmb)Co^III^(OH_2_) site from DFT showed metal-ligand bond lengths in agreement with crystal structures and our EXAFS data (Table 3). This structure also reproduced the small ctv feature of AqCbl^ox^, whereas an OH^-^ ligand yielded a too large ctv amplitude (Fig 5). The diminished ctv amplitude in AqCbl^red^ was best reproduced using a (dmb)Co^II^(OH_2_) site. Co(II) or Co(I) sites in which the water ligand, the dmb ligand, or both ligands were absent yielded larger ctv amplitudes and/or lower or higher peak energies disagreeing with the experimental data (Fig 5). A (dmb)Co^III^(CN) site reproduced the ctv feature of CNCbl^ox^ well. For CNCbl^red^, however, the increased ctv amplitude was only calculated for a Co^II^(CN) site (base-off), whereas (dmb)Co^II^(CN) or (dmb)Co^II^ sites yielded too small and shifted ctv features. The large ctv feature for MeCbl^ox^ was reproduced by the expected (dmb)Co^III^(CH_3_) geometry. This shows that the ctv feature is a specific indicator of cobalt redox and ligation changes.

**Table 3:** Cobalt-ligand bond lengths from crystallography, EXAFS, and DFT.

	species	bond length [Å]								
sample	(Lα)Co^x^(Lß)	Co-N_corrin_^mean^			Co-Lα			Co-Lß		
		crystal	EXAFS	DFT	crystal	EXAFS	DFT	crystal	EXAFS	DFT
AqCbl^ox^	(dmb)Co^III^(OH_2_)	1.89^a^	1.88	1.92	1.92^a^	1.92	1.96	1.95^a^	1.97	2.11
AqCbl^red^	(dmb)Co^II^(OH_2_)	-	1.89	1.92	-	2.30	2.36	-	2.47	2.96
	(dmb)Co^II^	-	1.89	1.92	-	2.30	2.30	-	-	-
CNCbl^ox^	(dmb)Co^II^(CN)	1.91^b^	1.89	1.92	2.04^b^	2.05	2.15	1.87^b^	1.85	1.88
CNCbl^red^	Co^II^(CN)	-	1.88	1.92	-			-	2.14	2.10
	(dmb)Co^II^	-	1.88	1.92	-	2.54	2.30	-	-	-
MeCbl^ox^	(dmb)Co^III^(CH_3_)	1.90^c^	1.89	1.92	2.16^c^	2.21	2.36	1.99^c^	1.95	1.97
CoFeSP-AqCbl^ox^	(OH_2_)Co^III^(OH_2_)	-	1.88	1.92	-	2.02	1.99	-	2.02	1.99
	Co^III^(OH_2_)	-	1.88	1.91	-	-	-	-	2.02	1.97
CoFeSP-AqCbl^red^	(OH_2_)Co^II^(OH_2_)	-	1.96	1.92	-	2.33	2.53	-	2.33	2.54
	Co^II^(OH_2_)	1.90^d^	1.86	1.91	-	-	-	2.55^d^	2.33	-
CoFeSP-AqCbl-RACo	Co^II^(O_Ser_)	1.90^e^	1.88	1.92	-	-	-	2.40^e^	2.10	2.08
CoFeSP-MeCbl^ox^	(OH_2_)Co^III^(CH_3_)	-	1.87	1.91	-	2.50	2.46	-	2.00	1.96
	Co^III^(CH_3_)	1.90^f^	1.87	1.91	-	-	-	2.00^f^	2.00	1.96

Crystal data for Cbl and CoFeSP-Cbl species were derived from refs.

^a^[56]

^b^[55]

^c^[54]

^d^[12, 19]

^e^[14]

^f^[18]

DFT data refer to geometry-optimized model structures with the indicated cobalt oxidation states and axial ligations; bond lengths from EXAFS (Table 2) were placed in the table to match the other data best and facilitate species comparison.

The small ctv feature of CoFeSP-AqCbl^ox^ was seemingly described by a 6-coordinated (OH_2_)Co^III^(OH_2_) site (Fig 5). However, the experimental ctv feature likely was increased by a Co(II) admixture so that a 5-coordinated Co^III^(OH_2_) site with a weak ctv feature at lower energies accounted equally well for the CoFeSP-AqCbl^ox^ spectrum. The broader and larger ctv feature of CoFeSP-AqCbl^red^ was best explained by dominance of the large ctv feature of a 5-coordinated Co^II^(OH_2_) site and minor contributions of weak ctv features from a Co(I) site without axial ligands (Fig 5). The large ctv peak at lower energies of CoFeSP-AqCbl-RACo was well reproduced assuming a Co^II^(O_Ser_) site, i.e. binding of the hydroxyl group of the serine of the RACo protein to cobalt at ß-position in the absence of an α-ligand, in agreement with the CoFeSP-RACo crystal structure [16]. Amplitude and energy of the largest ctv feature of CoFeSP-MeCbl^ox^ were reasonably reproduced only by a 5-coordinated Co^III^(CH_3_) site, whereas a (OH_2_)Co^III^(CH_3_) site showed a much too small ctv feature (Fig 5).

### Molecular structures of the cobalt sites

**Fig 6:**
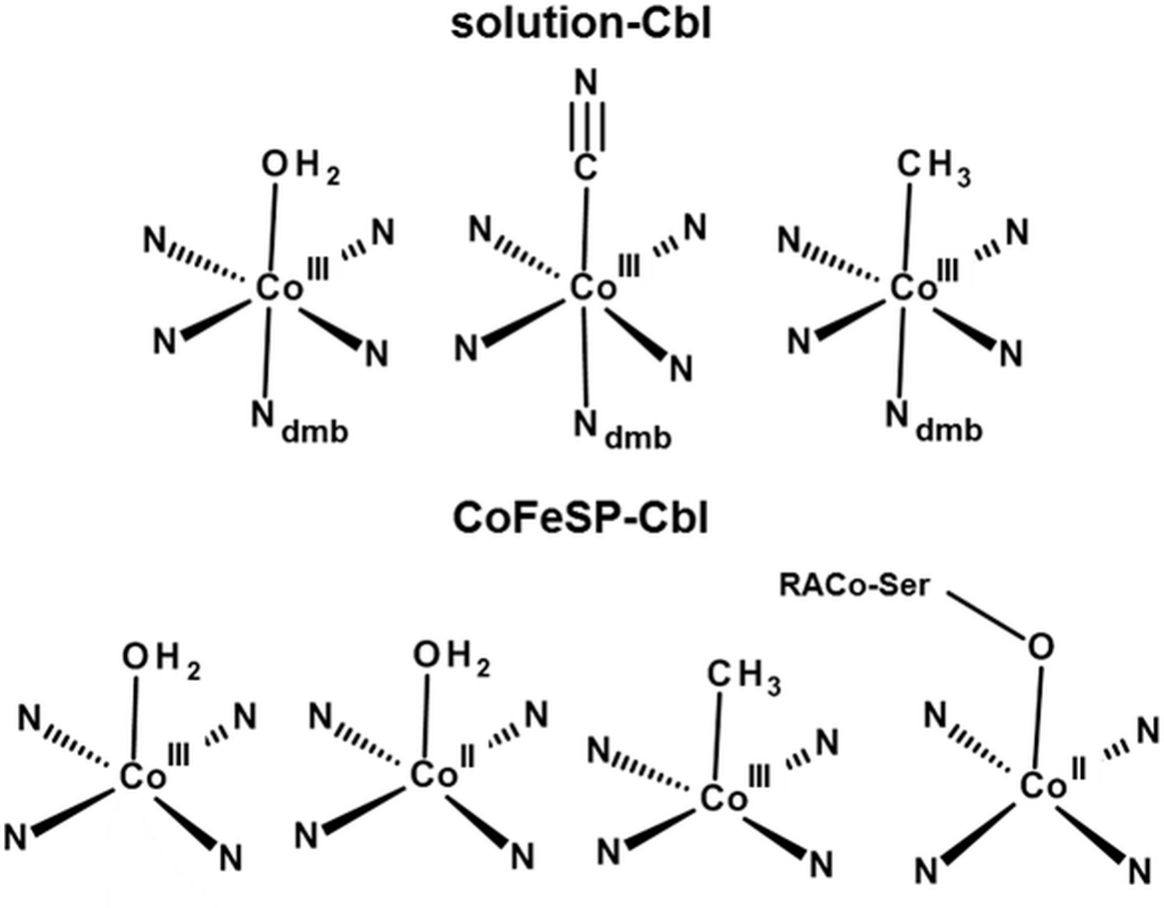
Cobalt coordination models. Shown structures represent most prominent species identified in the solution Cbl (top) and CoFeSP-Cbl (bottom) samples. The dmb ligand binds in α-position at cobalt; a water ligand in ß-position in Co(III)-containing CoFeSP-AqCbl cannot be fully excluded; RACo-Ser398 binds at Co(II) in the CoFeSP-RACo complex.

The analysis of the EXAFS, XANES, and ctv spectra using DFT (and multiple-scattering) calculations, as well as the TXRF, optical absorption, and EPR data, converged towards consistent cobalt site assignments (Fig 6). Solution Aq/CN/MeCbl samples showed the expected octahedral base-on (dmb)Co^III^(ß-ligand) configurations. AqCbl^red^ likely contained a (dmb)Co^II^(OH_2_) species with a weak water ligand (~2.5 Å) whereas CNCbl^red^ seemingly preferred a base-off Co^II^(CN) configuration with an elongated (~2.1 Å) Co-CN bond under our conditions. Co(III) species thus were generally 6-coordinated and Co(II) species preferred 6- or 5-coordinated geometries in solution Cbl.

The cobalt ion in CoFeSP-Cbl protein showed a tendency towards lower coordination numbers compared to the same metal oxidation state in solution Cbl. CoFeSP-AqCbl containing Co(III) presumably contained a 5-coordinated Co^III^(OH_2_) site as main species. Contributions from octahedral (OH_2_)Co^III^(OH_2_) sites, however, were not excluded. Single-electron reduction likely resulted in a Co^II^(OH_2_) site (Fig 6). CoFeSP-MeCbl^ox^ showed a clearly 5-coordinated Co^III^(CH_3_) site, meaning that water species at the α-position were undetectable. The cobalt spectral changes in the CoFeSP-RACo protein complex supported binding of the serine side chain of RACo to Co(II) at the ß-position (Fig 6).

### Electronic structure considerations

Calculated ctv spectra for the relevant low-spin cobalt site species were analyzed in terms of the electric dipole and quadrupole contributions to the underlying electronic transitions and of the metal/ligand characters of the target MOs. The ctv spectra were dominated (>75%) by formally selection-rule forbidden electric dipole transitions in 6-coordinated base-on and 5-coordinated base-off Co(III) and Co(II) sites with water ligands (Table 4). Increased contributions from allowed quadrupole transitions in the base-off sites lead to increased ctv intensities. Increased quadrupole contributions (up to ~50%) for Co(III) and Co(II) sites with two water ligands account for non-negligible ctv intensities in these symmetric structures. The ctv spectra of CH_3_-, CN-, or O_Ser_-ligand containing Co(III) sites showed almost exclusive dipole transitions, their more intense ctv features resulted from increased ligand characters of target MOs (Table 4). Dominating corrin character of target MOs for the corresponding Co(II) sites explained their more intense ctv features. The small contributions (<15%) of water ligands to target MOs generally exceeded those of dmb, but influenced the ctv intensities only moderately. However, twice as large ctv contributions from the methyl ligand and increased corrin contributions accounted for the large ctv features of the Co^III^(CH_3_)-containing sites. Similarly large corrin and weaker axial ligand contributions for the Co(II)(CN)/(O_Ser_) sites explained their smaller ctv features.

**Table 4:** Core-to-valence electronic transition characters.

	core-to-valence transition characters					
	electric contribution [%]^a^		metal/ligand contribution [%]^b^			
cobalt site	dipole	quadrupole	Co	corrin	Lα	Lß
(dmb)Co^III^(OH_2_)	85.0	11.3	58.3	25.8	4.2	11.7
(OH_2_)Co^III^(OH_2_)	61.1	38.1	46.5	47.9	2.8	2.8
Co^III^(OH_2_)	80.8	16.8	53.2	43.4	-	3.4
(dmb)Co^II^(OH_2_)	77.0	20.4	24.3	55.5	8.1	12.1
Co^II^(OH_2_)	74.8	23.5	26.9	58.4	-	14.7
(dmb)Co^III^(CH_3_)	96.5	0.3	28.0	24.2	15.5	32.3
Co^III^(CH_3_)	98.0	1.8	37.3	40.7	-	22.0
(dmb)Co^III^(CN)	88.7	9.8	35.5	49.4	7.2	7.9
Co^II^(CN)	93.7	5.6	40.3	53.8	-	5.9
Co^II^(O_Ser_)	93.1	6.1	41.9	54.4	-	3.7

Data represents the summed relative contributions to the respective DFT-calculated stick spectra underlying the ctv spectra in Fig 5.

^a^difference to 100% = magnetic pole contribution.

^b^Metal/ligand contributions (Lα, Lß = axial cobalt ligands) denote respective characters of ctv target molecular orbitals.

**Fig 7:**
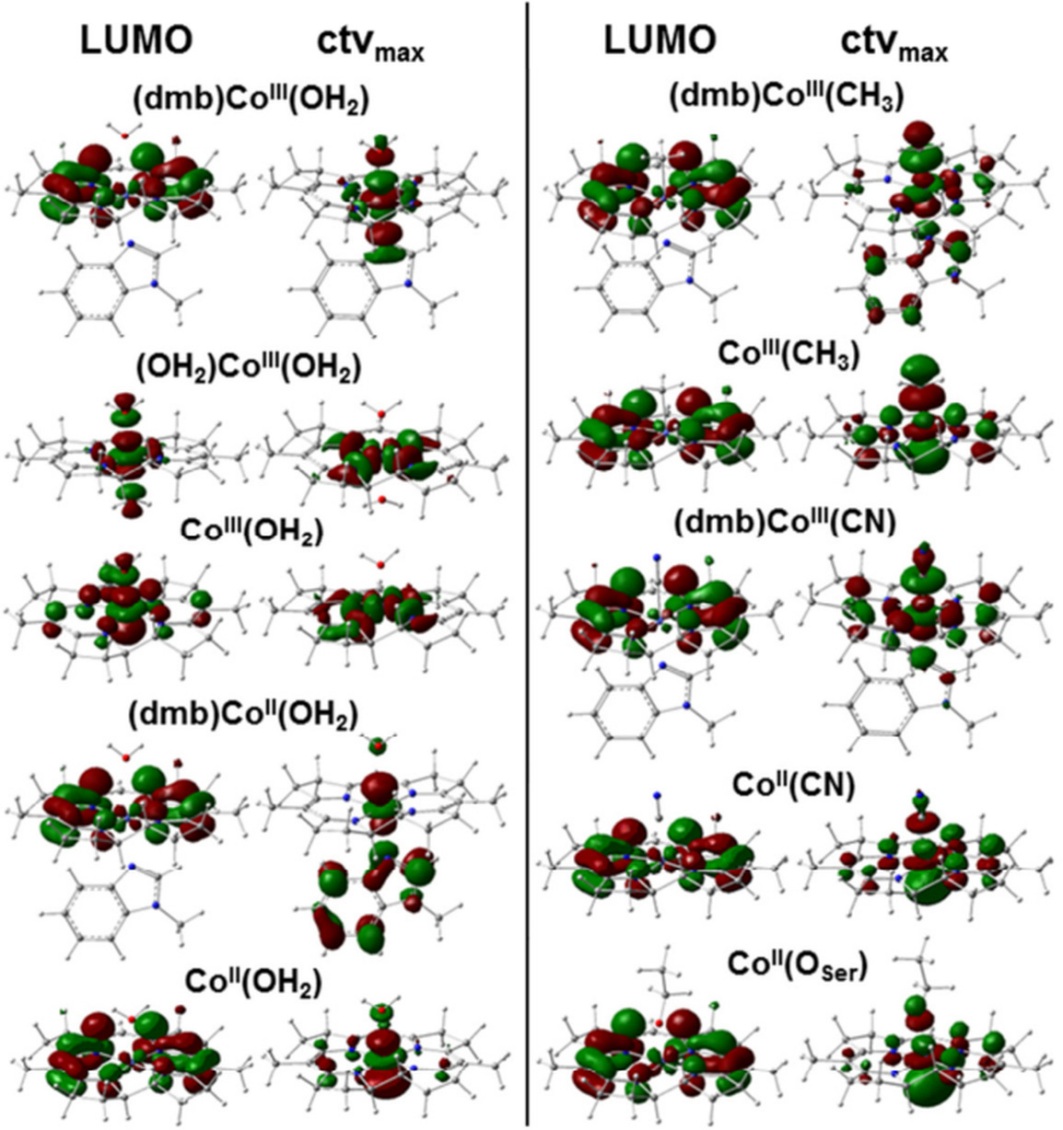
Molecular orbitals in Cbl model structures from DFT. LUMO, lowest unoccupied MO corresponding to the lowest energy core-to-valence electronic transition in the pre-edge absorption X-ray spectral region; ctv_max_, MO corresponding to the highest-intensity ctv transition of the pre-edge absorption. Cobalt oxidation state and axial ligation are indicated.

The LUMO, corresponding to the lowest-energy ctv transition, and the target MO for the maximal-intensity ctv transition were compared for the main cobalt site species (Fig 7). For (dmb)Co^III^(OH_2_) the LUMO was delocalized on the corrin ring and the highest-intensity target MO showed predominant Co-3*d*(z^2^) character oriented along the axial ligands. These MO locations were reversed when dmb was replaced by water. Enhanced delocalization of both orbitals over the corrin ring occurred in the absence of the α-ligand. Loss of the α-ligand further caused a 1–2 eV decrease of the HOMO and LUMO energies and a ~50% decrease of the LUMO–HOMO energy gap (ΔE) from ~3 eV in (dmb)Co^III^(OH_2_) to ~2 eV in Co^III^(OH_2_), mostly due to a larger relative E(LUMO) drop (Table 5). These energy changes are expected to facilitate reduction of Co^III^(OH_2_) at more positive potentials than (dmb)Co^III^(OH_2_). Exchange or loss of the Co(III) α-ligand further caused a cobalt charge increase by a factor up to ~1.5. For Co(II) species, less pronounced changes and LUMO delocalization onto the corrin rather independent of the α-ligand and more delocalized MOs with Co(*d*) character were found. However, a ~50% decreased ΔE compared to (dmb)Co^II^(OH_2_) was observed only for (OH_2_)Co^II^(OH_2_), due to a larger relative E(HOMO) drop, whereas Co^II^(OH_2_) showed an even slightly increased ΔE (Table 5). Compared to (dmb)Co^II^(OH_2_), (OH_2_)Co^II^(OH_2_) may thus be harder to reduce, but Co^II^(OH_2_) may be reduced at most positive potentials. The charge on cobalt for most Co(II) species was even slightly more positive compared to the Co(III) sites and the surplus negative charge was thus mostly located on the corrin ring.

**Table 5:** HOMO and LUMO energies and natural population analysis charges from DFT^a^.

cobalt site	energy [eV]		ΔE [eV]	NPA charge [e]			
	HOMO	LUMO		Co	corrin	L α	Lß
(dmb)Co^III^(OH_2_)	-11.4	-8.3	3.1	0.39	0.90	0.49	0.23
(OH_2_)Co^III^(OH_2_)	-11.8	-8.8	3.0	0.45	0.95	0.30	0.30
Co^III^(OH_2_)	-12.3	-10.2	2.1	0.56	1.15	-	0.28
(dmb)Co^II^(OH_2_)	-7.9 (-7.9)^b^	-4.5 (-4.5)^b^	3.4 (3.4)^b^	0.58	0.26	0.14	0.03
Co^II^(OH_2_)	-8.4 (-8.4)^b^	-4.8 (-4.8)^b^	3.6 (3.6)^b^	0.61	0.30	-	0.08
(dmb)Co^III^(CH_3_)	-7.9	-4.6	3.3	0.21	0.59	0.20	0.00
Co^III^(CH_3_)	-8.7	-5.0	3.7	0.30	0.65	-	0.05
(dmb)Co^III^(CN)	-8.2	-4.9	3.3	0.04	0.85	0.29	-0.18
Co^II^(CN)	-4.2 (-5.1)^b^	-1.8 (-1.8)^b^	2.4 (3.3)^b^	0.28	0.27	-	-0.55
Co^II^(O_Ser_)	-3.8 (-3.7)^b^	-1.6 (-1.6)^b^	2.2 (2.1)^b^	0.50	0.17	-	-0.67

^a^Values correspond to model structures with the indicated cobalt oxidation states and axial ligations in low-spin species

ΔE = E(LUMO)–E(HOMO); L α, Lß = axial cobalt ligands

^b^energies of “up" and “down” (in parenthesis) -spin MOs are given for the Co(II) species (and have the same energy in the Co(I) and Co(III) species).

For MeCbl, loss of the dmb ligand left the LUMO delocalization almost unchanged, but increased the valence level delocalization onto the corrin (Fig 7). Loss of the α-ligand rather increased ΔE due to a smaller relative E(LUMO) drop in Co^III^(CH_3_) compared to (dmb)Co^III^(CH_3_) (Table 5), making the Co^III^(CH_3_) species easier to reduce. In addition, the charges on the Co(III) center and in particular on the corrin in the MeCbl species were lowered compared to AqCbl, with little charge located on the methyl group. The MO configurations were remarkably similar in Co^II^(CN) and Co^II^(O_Ser_) (Fig 7), as were the similar E(HOMO/LUMO) and ΔE values. Significantly less positive charges on cobalt and corrin in Co^II^(CN) and in particular in Co^II^(O_Ser_) compared to the other Co(II) species were accompanied by strongly negative charges on the CN or O_Ser_ ligands. The relatively higher E(LUMO) compared, e.g., to Co^II^(OH_2_) suggested metal reduction at more negative potentials in Co^II^(O_Ser_), meaning that RACo binding to CoFeSP was expected to stabilize Co(II).

## Discussion

Molecular and electronic structures of cobalamin species bound to CoFeSP or in solution were characterized using XAS in combination with DFT calculations. The observed K-edge energies are affected both by axial coordination and formal cobalt oxidation state changes, in agreement with earlier studies [38, 39, 59]. Transition from octahedral cobalt sites in solution Cbl and reference compounds to square-pyramidal sites in CoFeSP-Cbl leads to relatively lower edge energies and significant shape changes, although the Co-N_corrin_ bond length shows only minor changes due to redox and geometry changes at cobalt. The EXAFS spectra were dominated by the Co-N_corrin_ bonds, but facilitated estimation of the axial ligand bond lengths, which were elongated for Co(II) species as in crystal and DFT structures.

The pre-edge absorption due to core-to-valence electronic excitations (ctv) revealed pronounced spectral variations in response to cobalt redox and site geometry changes. Interpretation of the ctv spectra in terms of resonant electronic excitation of a 1*s* core electron into unoccupied valence levels with variable metal/ligand characters was achieved using DFT. Good agreement between experimental and calculated ctv spectra was obtained for the solution Cbl and CoFeSP-Cbl systems, as previously found for other metal complexes (see, e.g., [30–33, 60–63]). The ctv intensity variations were consistently explained by changes in the cobalt/ligand character ratio of the target MOs and, to a lesser extent, by electric dipole/quadrupole contribution variations of the underlying electronic transitions due to axial ligation changes. This showed for example that the intense ctv features of cobalt sites with a methyl ligand are related to significant CH_3_ character of the target MOs, thus unambiguously establishing a Co^III^(CH_3_) site in CoFeSP-MeCbl. The ctv-XAS/DFT combination appears to be viable for redox state and ligation geometry assignment of cobalt sites in cobalamin.

The discriminated cofactor species revealed a trend for fewer ligands at cobalt in CoFeSP-Cbl compared to solution Cbl for the same oxidation state. Octahedral (dmb)Co^III^(OH_2_) and (dmb)Co^II^(OH_2_) sites were dominant in oxidized and reduced solution AqCbl. XAS and DFT showed a tendency for detachment of the water species from the Co(II) ion. However, the transition from (dmb)Co^III^(CN) to Co^II^(CN) species suggested preference for detachment of the weaker dmb ligand upon cobalt reduction for CNCbl in solution. MeCbl in solution showed the anticipated (dmb)Co^III^(CH_3_) structure. The spectroscopic and theoretical data converged to the same cobalt site structures in solution Cbl, corroborating the adequacy of the applied theory level (B3LYP/TZVP) for cobalamin structure description.

Crystal structures of CoFeSP-Cbl have shown the dmb group in base-off configuration in the protein [12, 14, 18, 19]. The crystal data furthermore were interpreted as showing the absence also of water species at the α-position. The oxygen of a threonine side chain (Thr374) at the α side was modeled at 3.2–4.6 Å to cobalt in different crystals (Fig 1), suggesting the absence of a Co-O_Thr_ bond. However, the ß-ligand bond length at cobalt also varied considerably or a ß-ligand was not assigned [12, 14, 18, 19]. These results could be related to site heterogeneity in the crystals, which may render detection of axial cobalt ligands difficult.

Our results suggest that oxidized CoFeSP-AqCbl contains mostly Co^III^(OH_2_) sites. Contributions from (OH_2_)Co^III^(OH_2_) sites, however, were not completely ruled out by our data. The presence or absence of a water ligand in the α-position at Co(III) in CoFeSP-AqCbl might be related for example to redox state variations of the [4Fe4S] cluster bound to the large subunit (CfsA) of CoFeSP [64–67]. Taking into account also the crystallographic data, we consider a Co^III^(OH_2_) site as more likely. Direct binding of Thr374 at the α-position at Co(III) was seemingly excluded, corroborating the crystallographic assignment. Single-electron reduction of CoFeSP-AqCbl results in formation of a Co^II^(OH_2_) site with a weaker water-cobalt interaction. Cobalt sites lacking a α-ligand were clearly identified in CoFeSP-MeCbl containing Co(III) and in CoFeSP-AqCbl-RACo containing Co(II) [18]. Our interpretation that a serine residue of RACo coordinates to Co(II) in CoFeSP-AqCbl-RACo is in agreement with the crystal structure [16] and previous spectroscopic data [15]. Serine binding at Co(II) likely is a prerequisite for ATP-induced CoFeSP activation and reaction with methyl-tetrahydrofolate, resulting in methyl group binding at cobalt [13, 14], as supported by our detection of a Co^III^(CH_3_) site in CoFeSP-MeCbl. In an earlier study of CoFeSP from another organism, a MeCbl species with a water (α) and a methyl (ß) ligand at Co(III) has been proposed; the water-cobalt bond, however, presumably was considerably elongated [65]. Our data favor a Co^III^(CH_3_) site in the *C*. *hydrogenoformans* enzyme under our conditions. We cannot fully exclude a remote water ligand, which might have escaped detection in the XAS analysis, but consider it as unlikely.

Our DFT calculations suggest that Co(III) and Co(II) reduction in base-off CoFeSP-AqCbl presumably occurs at more positive potentials compared to base-on AqCbl or CoFeSP-AqCbl with two water ligands. The determined reduction potentials of the Co(II/III) and Co(I/II) couples in CoFeSP-AqCbl (about +350 mV and -500 mV) indeed are more positive compared to the values of AqCbl in solution (about +200 mV and -600 mV) [4, 66, 68]. The absence of a α-ligand at cobalt thus may tune the Cbl reduction potential in CoFeSP into the physiological range for Co(I) formation prior to methylation [13, 18, 65]. However, our DFT results and redox titrations [14] suggest stabilization of the Co(II) state when Ser398 of RACo is bound to the metal. The resulting apparent disabling of Co(I) formation likely is overcome by ATP binding to the CoFeSP-RACo complex, inducing electron transfer from the [2Fe2S] cluster in RACo to the Co(II) site by a yet unresolved mechanism [14]. ATP binding could be accompanied by loss of the serine ligand at Co(II) to facilitate Co(I) formation by destabilizing Co(II), such that the Co(I/II) midpoint potential approaches the one of the [2Fe2S] cluster (-340 mV [14]). The square-planar Co(I) [36] then binds the methyl group to form Co^III^(CH_3_) and transfer of the methyl cation to acetyl-CoA synthase is facilitated by a more positive reduction potential, compared, e.g., to base-on MeCbl, of the Co^III^(CH_3_) site in CoFeSP. Control of the axial cobalt ligation therefore may play an important role both in methyl group transfer and reductive activation of CoFeSP.

## Conclusions

Analysis of cobalt K-edge XAS spectra in combination with DFT calculations of pre-edge absorption features facilitates determination of axial ligation and redox state of cobalt in solution Cbl and CoFeSP-Cbl. This supports the likely absence of a α-ligand in base-off CoFeSP-AqCbl and -MeCbl compared to base-on solution AqCbl and MeCbl, in agreement with earlier crystallographic and spectroscopic data. Coordination of a serine side chain from RACo to Co(II) in the CoFeSP-RACo protein complex is in agreement with our analysis. Control of the axial cobalt ligation may tune the redox potential of the cobalamin cofactor into the range of its electron transfer partners and likely is important for reductive activation of CoFeSP and methyl group shuttling.

## Supporting Information

S1 File optical absorption spectra of solution Cbl and CoFeSP-Cbl samples (Fig A), EPR spectra of CoFeSP-Cbl samples (Fig B), XANES spectra of cobalt reference compounds (Fig C), multiple scattering calculations of cobalamin XANES spectra (Fig D), K-edge energies from XANES simulations (Fig E), correlation of EXAFS fit parameters (Fig F), supporting references.
(PDF)

## Abbreviations

Cbl: cobalamin
CoFeSP: corrinoid-iron-sulfur-protein
ctv: core-to-valence electronic transitions
DFT: density functional theory
dmb: 5,6-dimethylbenzimidazole
EXAFS: extended X-ray absorption near edge structure
NaDT: sodium dithionite
RACo: reductive activator protein of CoFeSP
TiCi: titanium citrate
TXRF: total-reflection X-ray fluorescence analysis
XANES: X-ray absorption near edge structure
XAS: X-ray absorption spectroscopy
