# Supplementary material for: Axial Ligation and Redox Changes at the Cobalt Ion in Cobalamin Bound to Corrinoid Iron-Sulfur Protein (CoFeSP) or in Solution Characterized by XAS and DFT
Source: PLoS One. 2016 Jul 6;11(7):e0158681. doi: 10.1371/journal.pone.0158681 (PMC4934906; doi:10.1371/journal.pone.0158681)
Supplement: S1 File — optical absorption spectra of solution Cbl and CoFeSP-Cbl samples (Fig A), EPR spectra of CoFeSP-Cbl samples (Fig B), XANES spectra of cobalt reference compounds (Fig C), multiple scattering calculations of cobalamin XANES spectra (Fig D), K-edge energies from XANES simulations (Fig E), correlation of EXAFS fit parameters (Fig F), supporting references. (PDF) [file pone.0158681.s001.pdf]

Supporting Information

Axial ligation and redox changes at the cobalt ion in cobalamin bound to corrinoid iron-sulfur protein (CoFeSP) or in solution characterized by XAS and DFT

Peer Schrapers[1], Stefan Mebs[1], Sebastian Goetzl[2], Sandra E. Hennig[2], Holger Dau[1], Holger Dobbek[2], and Michael Haumann[1]

[1]Freie Universität Berlin, Department of Physics, 14195 Berlin, Germany

[2]Humboldt-Universität zu Berlin, Department of Biology, 10115 Berlin, Germany

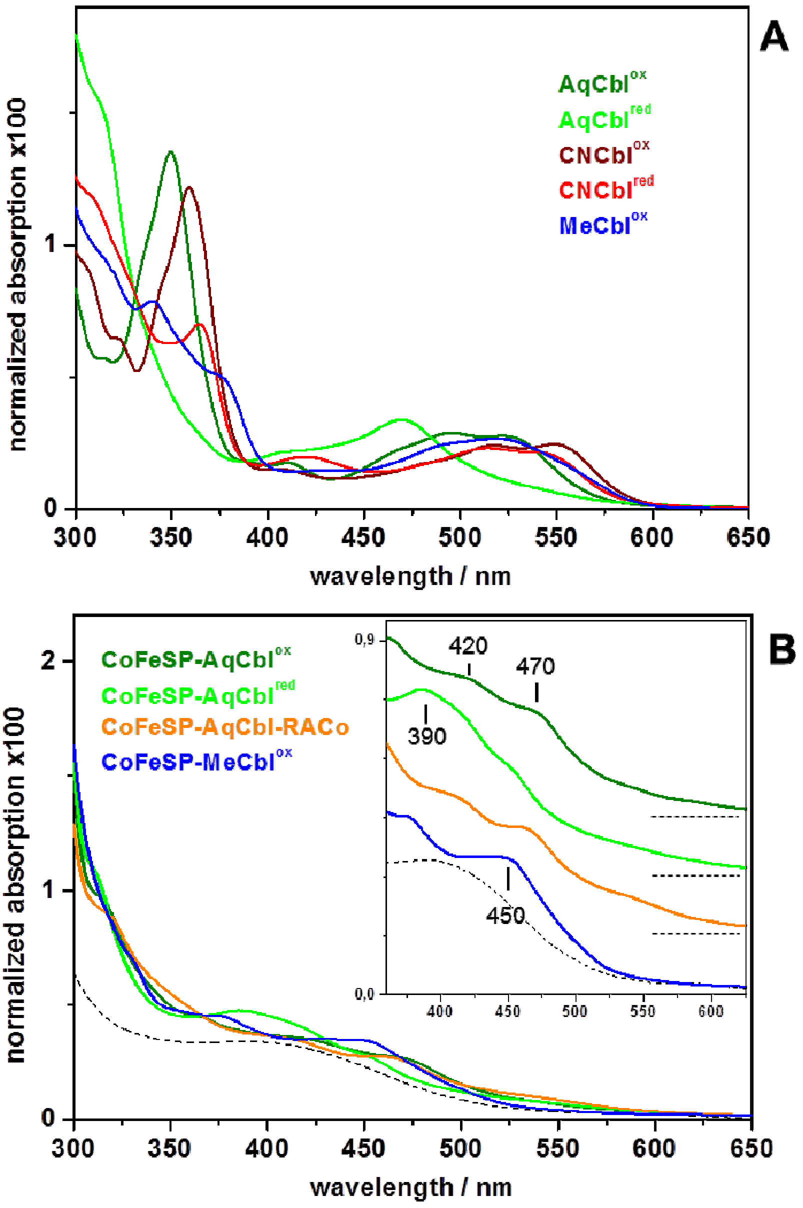

Figure A: Optical absorption spectra of Cbl and CoFeSP-Cbl samples. Spectra were normalized to unity area in the shown spectral range for comparison. **(A)** The spectra of the solution AqCbl^{ox}, CNCbl^{ox}, and MeCbl^{ox} samples are similar to spectra in the literature [1-5] and indicate the quantitative presence of base-on Co(III) sites in these samples. The pronounced peak at ~470 nm and the missing peak at ~360 nm (relative to AqCbl^{ox}) in the spectrum of AqCbl^{red} in comparison to spectra in the literature [1, 6-8], as well as the absence of a peak at ~390 nm typical for Co(I) in AqCbl [3, 9, 10] and missing in the AqCbl^{red} spectrum, indicate mostly Co(II) in the AqCbl^{red} sample. The spectrum of CNCbl^{red} resembles spectra of base-off Co(II)-containing Cbl species in the literature [11-14]. **(B)** The spectra of the CoFeSP-Cbl samples show absorption features due to the cobalamin cofactor in the 350-500 nm range, which are overlaid by spectral contributions from the proteins and the iron-sulfur clusters in CoFeSP and RACo [9, 15] (dotted black line, oxidized [4Fe4S] cluster and protein spectrum of CoFeSP lacking cobalamin [9]). Inset: CoFeSP spectra in magnification (vertically shifted). For CoFeSP-MeCbl^{ox}, the prominent peak at ~450 nm is indicative of Co(III) in the methyl-coordinated cobalt site [9, 15, 16]. For CoFeSP-AqCbl^{red}, the peak feature at ~390 nm indicates a minor Co(I) contribution [9, 15-19], comparison of the spectral amplitude ratios 450 nm : 390 nm [9, 15, 16] suggested ~30 % Co(I); the major species contained Co(II). In CoFeSP-AqCbl^{ox} the dominant species was Co(III) besides of a minor Co(II) contribution and in CoFeSP-AqCbl-RACo almost quantitative amounts of Co(II) were present, as revealed by EPR (see Fig B). A fully quantitative determination of Co(I), Co(II), and Co(III) contents in the CoFeSP/RACo samples using UV/vis spectra is difficult due to varying FeS cluster contributions [9, 15, 19].

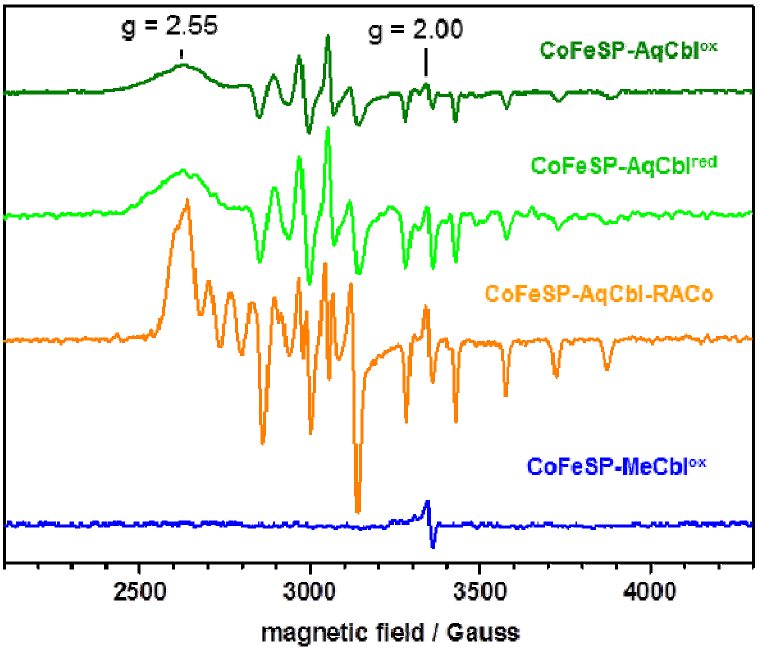

Figure B: EPR spectra of CoFeSP-Cbl samples. Co(II) signals in oxidized and reduced CoFeSP samples were vertically shifted. Co(II) spins per cobalt ion (Table 1) were determined relative to the signal of a $Cu^{II}SO_4$ solution spin-standard with known concentration (0.4 mM) measured with the same instrument settings and using double-integration of signals for area determination and the Co concentrations in the samples determined by TXRF (Table 1). Shown traces were normalized to the relative Co(II) spin counts for comparison. The signal of CoFeSP-AqCbl is indicative of the base-off configuration of the cofactor; note the narrowing of the g_x line around g = 2.55 in the presence of RACo, which has been interpreted as reflecting the binding of a serine residue of RACo to the Co(II) ion in CoFeSP, which is visible in the crystal structure [20, 21]. Similar EPR spectra of Co(II) species in CoFeSP and CoFeSP-RACo have been analyzed previously in detail [20, 21] (see also [15, 22, 23]). EPR spectra at X-band (9.39 GHz) were recorded using the previously described EPR spectrometer equipped with a liquid-He cryostat [24] with the following instrument settings: temperature, 80 K; microwave power, 2 mW; modulation frequency, 100 kHz; modulation amplitude, 5 Gauss. Spectra were corrected for the cavity background by subtracting spectra of buffer-filled EPR quartz tubes and smooth spline curves for residual baseline adjustment.

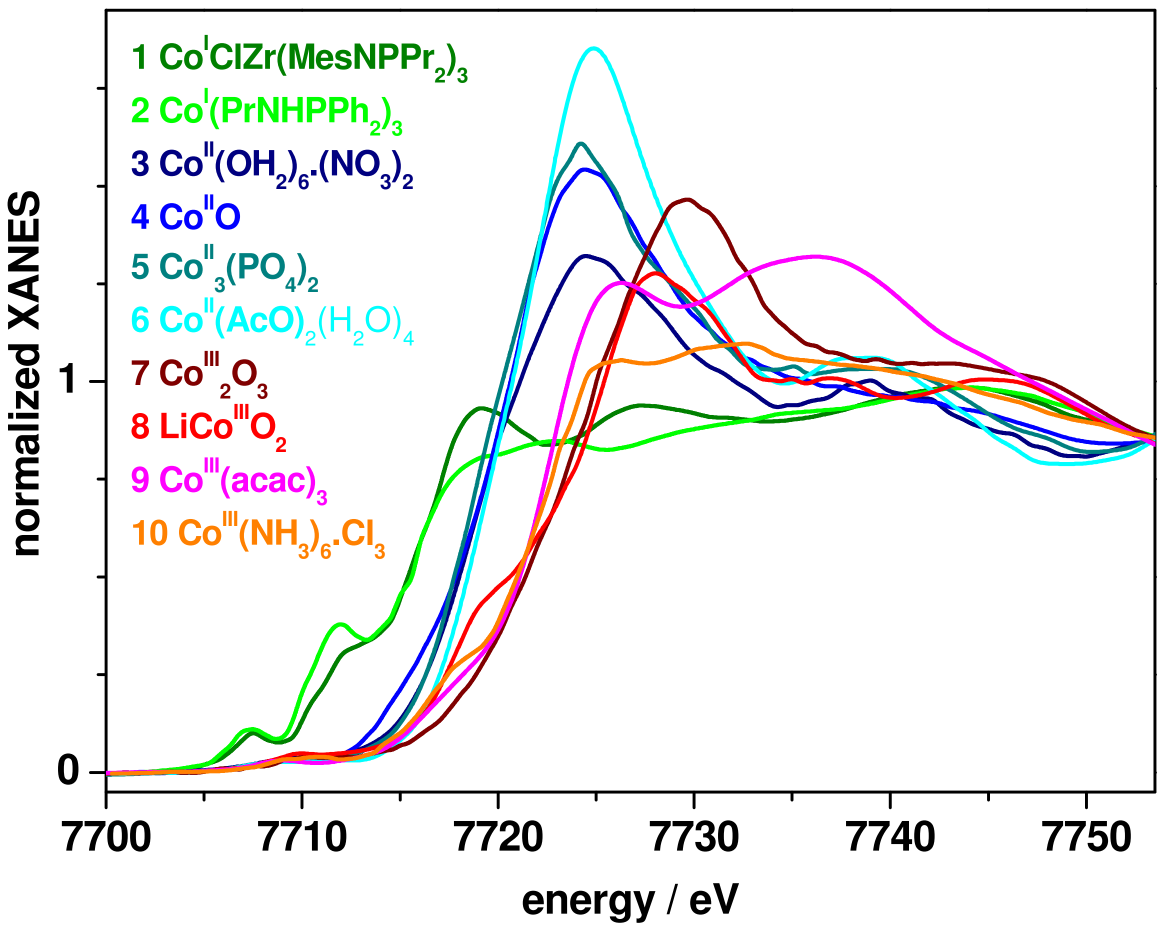

Figure C: XANES spectra of cobalt reference compounds. Spectra labelled **1-10** correspond to the following references: **1**, **2**, and **9** [25], spectra were reproduced from figure 1 in the reference (**1** and **2**) and figure S2 (**9**) in the Supporting Information of the reference; **3** and **10** [26], spectra were reproduced from figure 1 in the reference; **4-8**, this work. Cobalt oxidation states are indicated. K-edge energies of the spectra were determined at 50 % level of the normalized XANES (see Fig 4). The ~1 eV edge energy spread for a given cobalt redox state (Fig 4) reflected the K-edge shape changes in response to metal ligation changes in the reference compounds, with the complexes including more strong-field ligands and higher coordination numbers in tendency showing the highest K-edge energies.

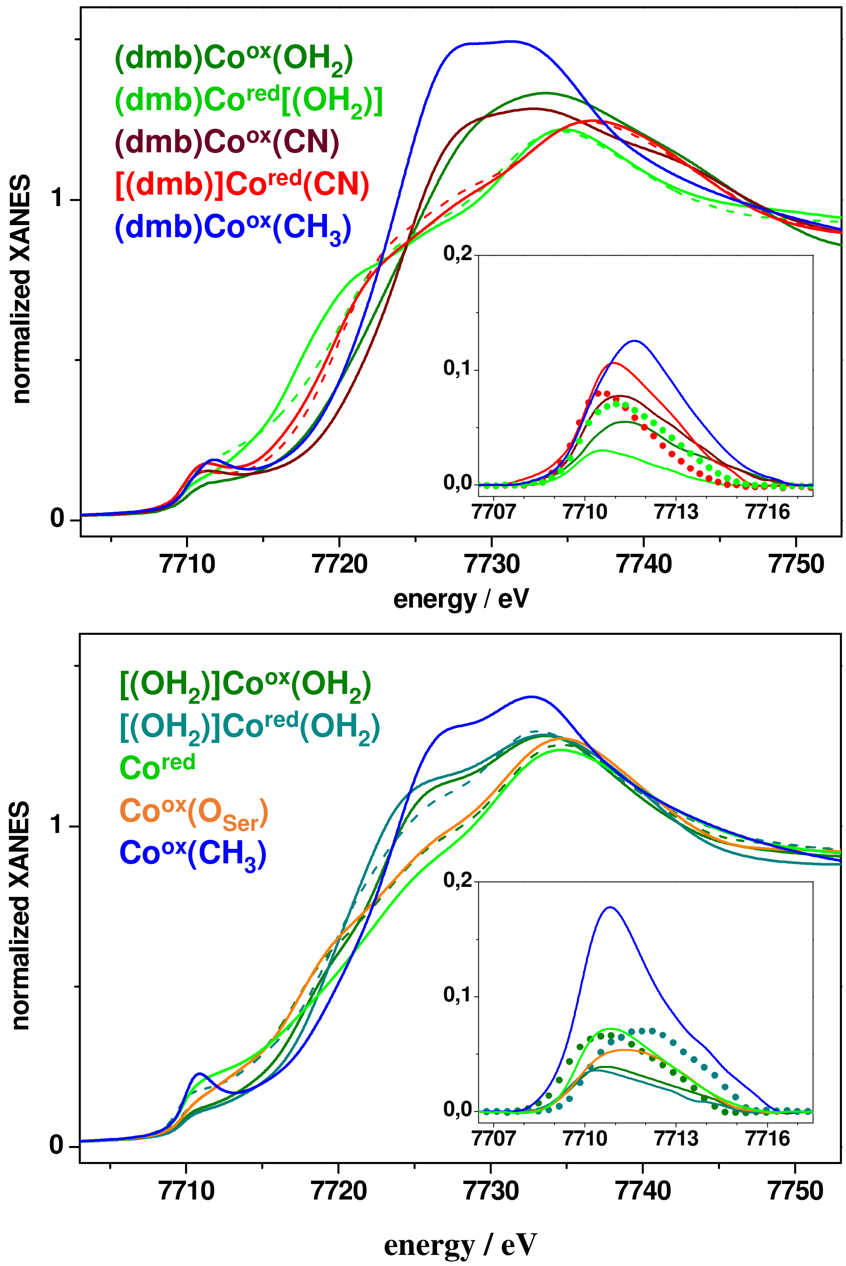

Figure D: Qualitative multiple-scattering calculations of cobalamin XANES spectra. Spectra were calculated using the FEFF9.0 code [27] with the full-multiple-scattering (FMS) and self-consistent-field (SCF) options activated and using model structures (based on crystal structure 2H9A in the PDB, 1.9 Å resolution and constructed using the program Hyperchem) consisting of a truncated corrin ring and (where present) a truncated dimethylbenzimidazole (dmb) group (compare Fig 7) and the indicated axial ligand species, i.e. (Lα)Co(Lβ). The distances between cobalt and the axial ligands were set in the structures to the values derived from our EXAFS analysis (Table 1) and the orientation of the ligands was roughly optimized using the Hyperchem program. The annotations of spectra refer to the cobalt-ligand bond lengths determined for the (initially present) Co(III) species (*ox*) and the reduced (i.e. Co(II)/Co(I) containing) species (*red*). Dashed lines (and circles in the inset) correspond to simulations, in which the cobalt ligands in brackets in the annotations were absent. The serine side chain was represented by an ethyl group in the Co(O_{Ser}) calculation The Co-N/C_{corrin} distances were kept at the crystallographic values in all calculations to emphasize the influence of the axial ligands on the XANES shape (the cobalt redox state is not explicitly considered in the calculations). Calculated spectra were shifted by -3.55 eV on the energy axis for comparison with experimental data (see Fig E). Inset: isolated pre-edge features obtained after removal of the background due to the main edge rise using a smooth polynomial spline curve.

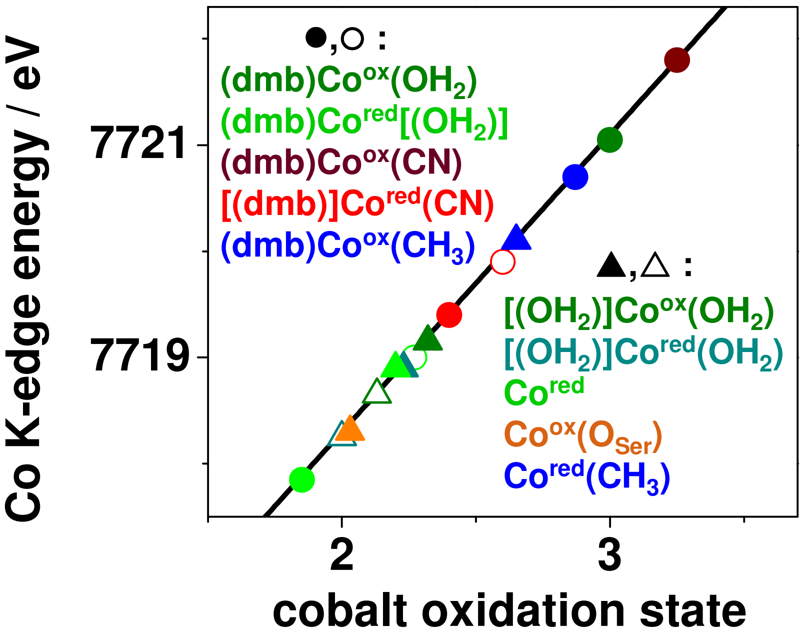

Figure E: K-edge energies from XANES simulations. K-edge energies of XANES spectra were derived from multiple-scattering theory calculations on model structures for the cobalamin systems (Fig D) and are placed on the same fit curve as shown in Fig 4 (symbols correspond to the indicated axial cobalt ligations and redox states; open symbols correspond to the respective structures lacking the axial ligand in brackets in the annotation). The experimental ~2.8 eV edge energy downshift for $AqCbl^{red}$ compared to $AqCbl^{ox}$ was best matched using a (dmb)Co(OH_2) site with ~2.3 Å Co-N and ~2.5 Å Co-O distances whereas a β-ligand lacking structure yielded a ~1 eV higher edge energy than experimentally observed (Fig 4). For $CNCbl^{red}$, the large edge energy decrease compared to $CNCbl^{ox}$ was better reproduced by a base-off cobalt site retaining the cyanide ligand than by base-on sites with or without the CN^- ligand showing higher edge energies (Fig D).

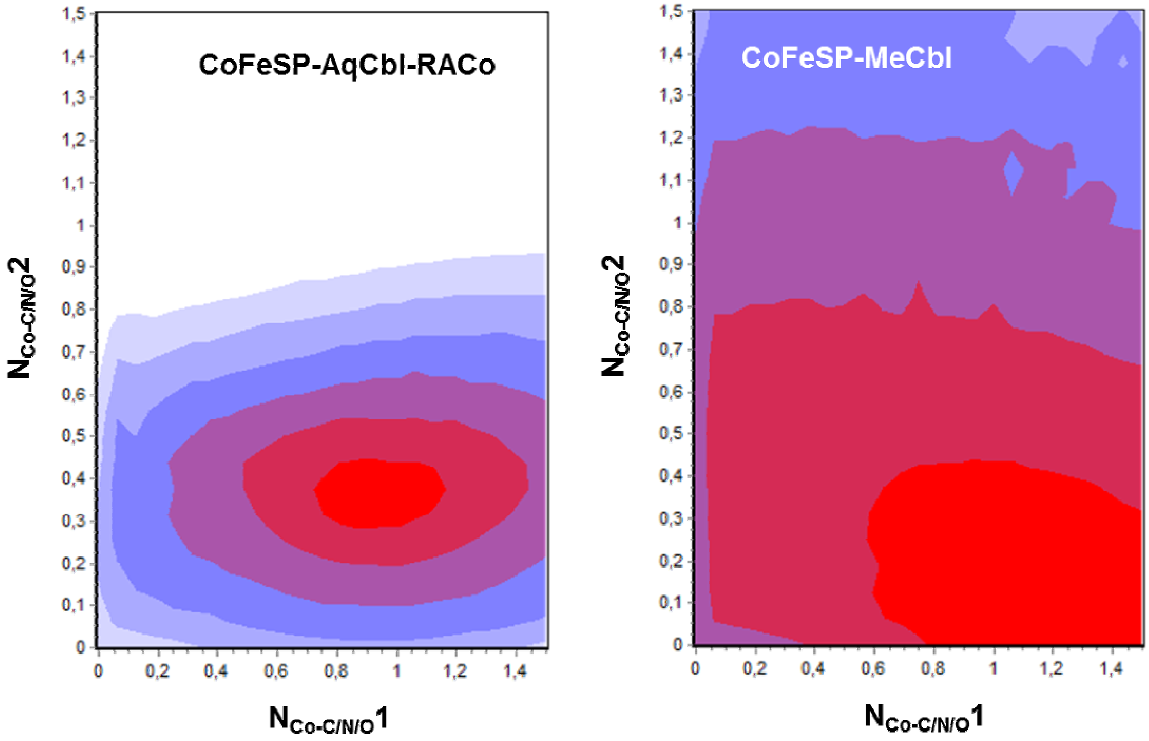

Figure F: Correlation of EXAFS fit parameters. Shown are plots of EXAFS fit results for CoFeSP-AqCbl-RACo and CoFeSP-MeCbl samples for variation of coordination numbers (N) of the Co-C/N/O shell 1 (R of about 2.1 Å or 2.0 Å) versus the Co-C/N/O shell 2 (R ~ 2.5 Å) (see Table 2, fits 19 and 21). Light-blue to red colors denote increasing fit quality (red color = lowest R_F value). The correlation plots support the low coordination number of the second Co-C/N/O distance and therefore predominance of 5-coordinated Co(II) or Co(III) sites in the samples.

Supporting References

1. Salnikov DS, Silaghi-Dumitrescu R, Makarov SV, van Eldik R, Boss GR. Cobalamin reduction by dithionite. Evidence for the formation of a six-coordinate cobalamin(II) complex. Dalton Trans. 2011;40(38):9831-4.
2. Santoro G, Zlateva T, Ruggi A, Quaroni L, Zobi F. Synthesis, characterization and cellular location of cytotoxic constitutional organometallic isomers of rhenium delivered on a cyanocobalmin scaffold. Dalton Trans. 2015;44(15):6999-7008.
3. Achey D, Brigham EC, DiMarco BN, Meyer GJ. Excited state electron transfer after visible light absorption by the Co(I) state of vitamin B12. Chem Commun (Camb). 2014;50(87):13304-6.
4. Eisenberg AS, Likhtina IV, Znamenskiy VS, Birke RL. Electronic spectroscopy and computational studies of glutathionylco(III)balamin. J Phys Chem A. 2012;116(25):6851-69.
5. Kruszyna H, Magyar JS, Rochelle LG, Russell MA, Smith RP, Wilcox DE. Spectroscopic studies of nitric oxide (NO) interactions with cobalamins: reaction of NO with superoxocobalamin(III) likely accounts for cobalamin reversal of the biological effects of NO. J Pharmacol Exp Therap. 1998;285(2):665-71.
6. Grodkowski J, Neta P. Cobalt corrin catalyzed photoreduction of CO2. J Phys Chem A. 2000;104(9):1848-1853.
7. Harris DA, Stickrath AB, Carroll EC, Sension RJ. Influence of environment on the electronic structure of Cob(III)alamins: time-resolved absorption studies of the S(1) state spectrum and dynamics. J Am Chem Soc. 2007;129(24):7578-85.
8. Dassanayake RS, Shelley JT, Cabelli DE, Brasch NE. Pulse radiolysis and ultra-high-performance liquid chromatography/high-resolution mass spectrometry studies on the reactions of the carbonate radical with vitamin B12 derivatives. Chemistry. 2015;21(17):6409-19.
9. Goetzl S, Jeoung JH, Hennig SE, Dobbek H. Structural basis for electron and methyl-group transfer in a methyltransferase system operating in the reductive acetyl-CoA pathway. J Mol Biol. 2011;411(1):96-109.
10. Lexa D, Saveant JM, Zickler J. Electrochemistry of vitamin-B12 .5. cyanocobalamins. J Am Chem Soc. 1980;102(8):2654-2663.
11. Sagi I, Wirt MD, Chen EF, Frisbie S, Chance MR. Structure of an Intermediate of Coenzyme-B12 Catalysis by Exafs - Cobalt(Ii)-B12. J Am Chem Soc. 1990;112(24):8639-8644.
12. Mannel-Croise C, Zelder F. Immobilised vitamin B12 as a biomimetic model for base-off/histidine-on coordination. Chem Commun. 2011;47(40):11249-11251.
13. Kim J, Gherasim C, Banerjee R. Decyanation of vitamin B12 by a trafficking chaperone. Proc Natl Acad Sci U S A. 2008;105(38):14551-4.
14. oProinsias K, Giedyk M, Gryko D. Vitamin B12: chemical modifications. Chem Soc Rev. 2013;42(16):6605-19.
15. Menon S, Ragsdale SW. Role of the [4Fe-4S] cluster in reductive activation of the cobalt center of the corrinoid iron-sulfur protein from *Clostridium thermoaceticum* during acetate biosynthesis. Biochemistry. 1998;37(16):5689-98.
16. Tan XS, Sewell C, Lindahl PA. Stopped-flow kinetics of methyl group transfer between the corrinoid-iron-sulfur protein and acetyl-coenzyme A synthase from *Clostridium thermoaceticum*. J Am Chem Soc. 2002;124(22):6277-84.
17. Kung Y, Ando N, Doukov TI, Blasiak LC, Bender G, Seravalli J, et al. Visualizing molecular juggling within a B12-dependent methyltransferase complex. Nature. 2012;484(7393):265-9.

18. Menon S, Ragsdale SW. The role of an iron-sulfur cluster in an enzymatic methylation reaction. Methylation of CO dehydrogenase/acetyl-CoA synthase by the methylated corrinoid iron-sulfur protein. J Biol Chem. 1999;274(17):11513-8.
19. Hennig SE, Jeoung JH, Goetzl S, Dobbek H. Redox-dependent complex formation by an ATP-dependent activator of the corrinoid/iron-sulfur protein. Proc Natl Acad Sci U S A. 2012;109(14):5235-40.
20. Meister W, Hennig SE, Jeoung JH, Lendzian F, Dobbek H, Hildebrandt P. Complex formation with the activator RACo affects the corrinoid structure of CoFeSP. Biochemistry. 2012;51(36):7040-2.
21. Hennig SE, Goetzl S, Jeoung JH, Bommer M, Lendzian F, Hildebrandt P, et al. ATP-induced electron transfer by redox-selective partner recognition. Nat Commun. 2014;5:4626.
22. Stich TA, Seravalli J, Venkateshrao S, Spiro TG, Ragsdale SW, Brunold TC. Spectroscopic studies of the corrinoid/iron-sulfur protein from *Moorella thermoacetica*. J Am Chem Soc. 2006;128(15):5010-20.
23. Jablonski PE, Lu WP, Ragsdale SW, Ferry JG. Characterization of the metal centers of the corrinoid/iron-sulfur component of the CO dehydrogenase enzyme complex from *Methanosarcina thermophila* by EPR spectroscopy and spectroelectrochemistry. J Biol Chem. 1993;268(1):325-9.
24. Lowenstein J, Lauterbach L, Teutloff C, Lenz O, Bittl R. Active site of the NAD(+)-reducing hydrogenase from *Ralstonia eutropha* studied by EPR spectroscopy. J Phys Chem B. 2015;119(43):13834-41.
25. Krogman JP, Gallagher JR, Zhang G, Hock AS, Miller JT, Thomas CM. Assignment of the oxidation states of Zr and Co in a highly reactive heterobimetallic Zr/Co complex using X-ray absorption spectroscopy (XANES). Dalton Trans. 2014;43(37):13852-13857.
26. Risch M, Khare V, Zaharieva I, Gerencser L, Chernev P, Dau H. Cobalt-oxo core of a water-oxidizing catalyst film. J Am Chem Soc. 2009;131(20):6936-6937.
27. Rehr JJ, Kas JJ, Vila FD, Prange MP, Jorissen K. Parameter-free calculations of X-ray spectra with FEFF9. Phys Chem Chem Phys. 2010;12(21):5503-13.
